# Proteomic study for the prediction of μCT imaging with iodine

**DOI:** 10.1007/s00418-026-02532-3

**Published:** 2026-09-25

**Authors:** Valentin Wesp, Heiko Stark, Lisa-Marie Barf

**Affiliations:** 1 Matthias Schleiden Institute, Friedrich Schiller University, Inselplatz 5, 07743 Jena, Thuringia Germany; 2 Institute of Zoology and Evolutionary Research with Phyletic Museum, Friedrich Schiller University, Erbertstr. 1, 07743 Jena, Thuringia Germany

**Keywords:** Human proteome, Skin, Mucosa, Skeletal muscle, Collagen

## Abstract

Iodine-based staining techniques are commonly used in histological imaging and micro-computed tomography (μCT) because iodine binds to specific molecules. However, the basis for tissue-specific contrast has not yet been sufficiently explored. In this study, we analysed the human proteome at four levels: individual proteins, protein families, tissues with additional expression values for selected proteins, and organs as distinct combinations of tissues. At each level, we identified groups with high potential for iodine binding, particularly those rich in aromatic heterocyclic amino acids. We evaluated the occurrence of aromatic and non-aromatic heterocyclic and carbocyclic amino acids in 20,650 proteins, 1487 protein families, 57 tissues, and 16 organs using bioinformatic methods. This evaluation was based on curated human protein sequences and integrated expression information from the Human Protein Atlas and ProteomicsDB. At the protein level, structural proteins such as titin, nebulin, obscurin, mucin, filaggrin, and hornerin exhibited a high absolute number of aromatic heterocyclic amino acids, which could explain the high μCT contrast in muscle, skin, and mucosal tissues. A smaller set of proteins displayed strong relative enrichment. At the family level, the average absolute values were highest in families containing very large proteins. However, relative values highlighted sequence-level enrichment in selected enzyme- and membrane-associated families. In contrast, tissue- and organ-level enrichment showed only small, non-significant differences depending on the expression dataset, and was highly similar across groups. Furthermore, enrichment was not robustly associated with iodine staining intensity in organs. Our results provide a foundation for iodine-based tissue imaging and could serve as a starting point for future research, such as cross-species applications and the structural and functional effects of iodination.

**Supplementary Information:**

The online version contains supplementary material available at https://doi.org/10.1007/s00418-026-02532-3.

## Introduction

Iodine has been used in histology for tissue staining for almost two centuries. The most common and least costly form is Lugol’s solution, which is a dissolved mixture of iodine and potassium iodide. It was first described by Jean Guillaume Auguste Lugol in the early 1800s (Fennerty 1999). In solution, iodine (I2) and potassium iodide (KI) additionally form triiodides (I3$^{+}$) and potassium ions (K$^{-}$) (Degenhardt et al. 2010). This solution causes the normal, non-cornified squamous epithelium of the healthy oesophagus, for example, to discolour differently from pathologically altered tissue (Fennerty 1999; Canto 1999). This is explained by the affinity of glycogen for iodine-containing substances (Bock and Shear 1972; Metscher 2009a). Lipid staining is also known (Gignac and Kley 2014; Gignac et al. 2016). The contrast mechanism for high-contrast computed tomography (CT) imaging at various levels of resolution (organs, tissues, proteins) is not yet fully understood (Metscher 2009a).

It is known that pure iodine is an inert halogen that usually requires catalysis to form a bond (Ajvazi and Stavber 2022). However, it can easily form non-covalent bonds, including oxidation of disulfide bridges (Cys) and hydrophilic and ion–ion interactions (amino and carbonyl groups) (Clark et al. 2005; Politzer et al. 2007; Cavallo et al. 2016). Another possibility of staining can be explained by intercalation processes into helical structures of starch with triiodide, leading to an intense blue-black colour (Mulliken 1950; Pesek et al. 2022; Pesek and Silaghi-Dumitrescu 2024). Electron-rich aromatic heterocycles (pyrrole, furan, or thiophene), on the other hand, can react very easily directly with iodine, called iodination (Stavber et al. 2008). Such aromatic heterocyclic compounds are a widespread class in nature, with important roles in biological processes. They include the nucleic bases of RNA and DNA, cytochromes, cobalamins, some natural colourants and odorants, and the amino acids histidine and tryptophan (Vollhardt and Schore 2020; Schuster and Malycheva 2025). Proline, as a saturated five-membered heterocycle, does not follow Hückel’s rules and is thus not aromatic. Therefore, it does not form a halogen bond with pure iodine.

Two reviews by Metscher (2009a, 2009b) show the strength of iodine staining. He compares several contrast agents (gallocyanin-chromalum, I2E, I2M, IKI, PTA, osmium tetroxide) for the staining of soft tissue. There are several other studies on this subject (Orellana et al. 2024; de Bournonville et al. 2019; Norris et al. 2013; Jacobs et al. 2024; Dawood et al. 2021). Iodine provides results comparable to those of the other contrast agents. However, it is preferable in terms of usability and toxicity (Metscher 2009b). It has also been shown that iodine primarily stains yolk, skin, eyes, nerves, and muscle tissue, which are protein-rich tissues.

There is also a special analysis method, called the iodine value determination, that measures the degree of unsaturated compounds in fats (Thomas 2000). This utilizes the fact that interhalogens (iodine chloride) specifically add to olefinic bonds and can be used for quality control. However, it should be noted that the iodine has to be bonded; otherwise, it will not attach to the alkenes. This method also explains why iodine can be used to stain lipids.

So why is iodine used to stain soft tissue for micro-computed tomography (μCT) examinations? This is because soft tissue has a largely homogeneous density and offers little contrast compared with mineralized tissues such as bone. The reason for this is that soft tissue consists mainly of water, which is the primary signal in μCT and therefore masks the signals from other components (proteins, fatty acids, etc.). Iodine-based contrast is able to amplify the signals from these components. Iodine helps here because of its atomic radius. The radius of a covalently bonded iodine atom is around 133 picometres, which falls within the wavelength range of X-rays: 10–10,000 picometres (Dean and Lange 1999). X-rays are therefore easily absorbed by iodine atoms and are thus suitable for enhancing contrast. Furthermore, as iodine rarely occurs naturally in animal tissue, it is suitable for the specific visualization of iodine-affine organs, tissues, and proteins.

On the basis of the above-mentioned facts, aromatic heterocycles and olefinic double bonds can be selectively stained and spatially visualized through CT techniques. This raises the question of which potential metabolites or proteins are highlighted by iodine-based contrast. Since iodine can easily bind to aromatic heterocycles (histidine and tryptophan), it can be expected that proteins with high content of these amino acids can be stained with iodine particularly well. However, these amino acids imply high metabolic costs in their synthesis. As enzymes and regulatory proteins need a wide diversity of amino acids while structural proteins do not, a working hypothesis is that the former are rich in aromatic amino acids, in contrast to the latter. We will check this hypothesis in the present paper.

## Methods

For our study, we retrieved the human proteome (containing 20,650 proteins) in FASTA format from the UniProt database (accessed 22 June 2026) using a custom Python script (v3.14). We chose a human dataset as a proof of concept due to its high level of curation and relevance. After filtering for uncurated sequences, 322 were removed, leaving 20,328 curated sequences in our dataset. The remaining sequences were then analysed for their amino acid composition, quantified as both absolute counts and proportions of aromatic residues (histidine and tryptophan), non-aromatic heterocyclic residues (proline), aromatic carbocyclic residues (phenylalanine and tyrosine), and all other canonical amino acids.

To compare specific staining in μCT, intensities and expression values for the aforementioned proteome were downloaded from three selected databases: the MorphoSource Database (MS), the Human Protein Atlas (HPA), and the ProteomicsDB (PtDB) (Schmidt et al. 2018; Samaras et al. 2020).

**Fig. 1:**
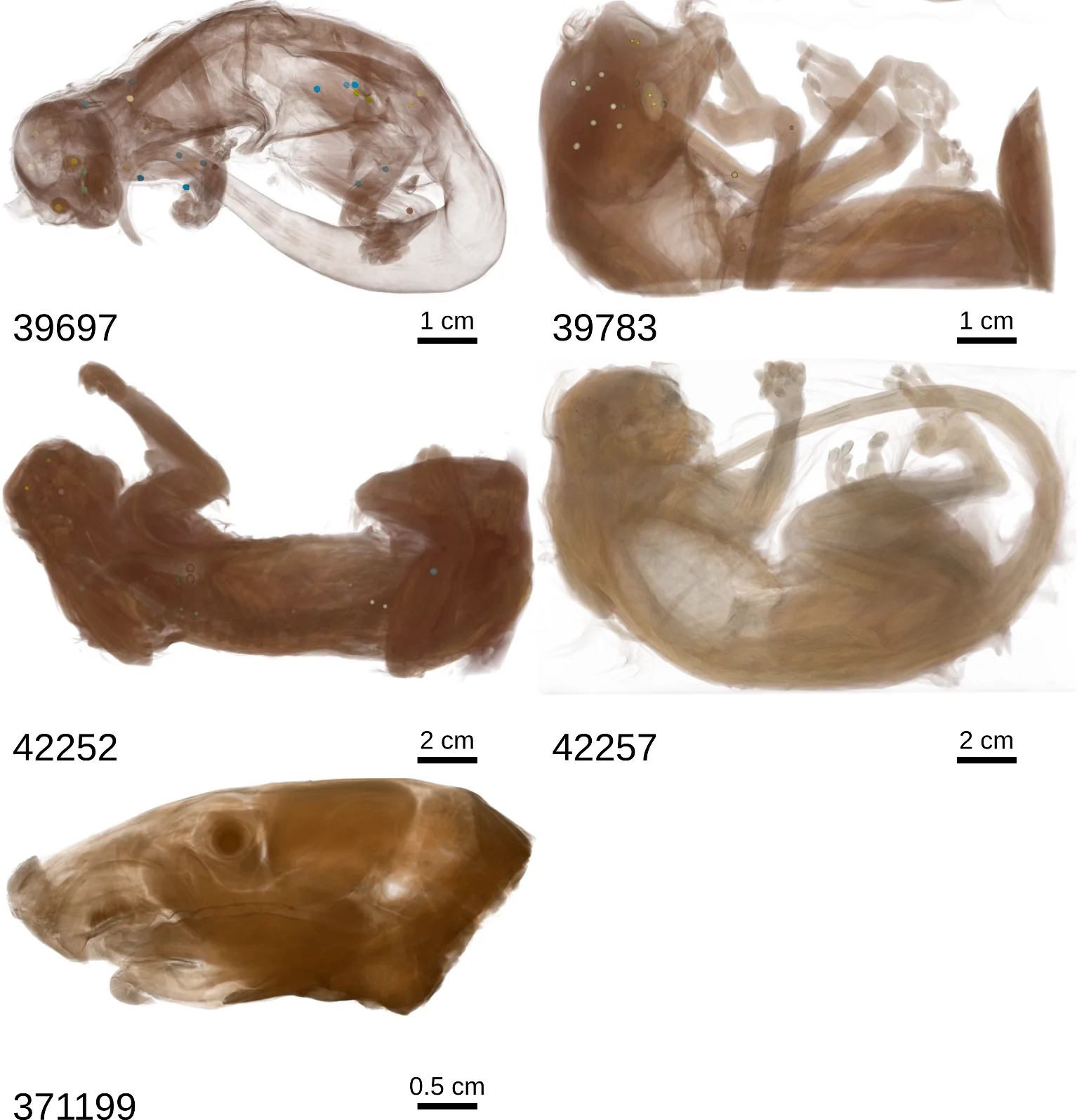
Overview of the datasets enriched with iodine-based contrast agents from the MorphoSource database and the local sampling sites for these five mammals (MorphoSource: 000039697—fat-tailed dwarf lemur, 000039783—red slender loris, 000042252—West African potto, 000042257—northern giant mouse lemur, 000371199—rat’s head). Please note that the skin and most internal organs were removed to improve diffusion, so not all organs could be analysed in all animals

For the MS database, this includes iodine-stained volume data for five mammalian species (000039697, 000039783, 000042252, 000042257, 000371199) (Yapuncich et al. 2019). Differentiating tissues was not possible due to the low resolution compared with normal histological staining. Therefore, the volume data were restricted to organs. The analysis was performed using 3D Slicer to obtain normalized intensity values for each available organ. It should be noted that not all organs were present in all animals, as some had been removed to improve iodine contrast (Fig. 1).

Next, we grouped proteins into four levels to obtain distinct enrichment values for selected amino acid groups: protein, family, tissue, and organ. For the protein level, enrichment was simply calculated as the absolute and relative proportions of selected residues in each protein. For the family level, proteins were grouped based on the HUGO Gene Nomenclature Committee (HGNC) database, which contains 1447 families. Of the 20,328 proteins in the dataset, 4760 could not be assigned to any family. For this level, enrichment was calculated as the average of absolute and relative proportions of selected residues of each protein in a family.

**Fig. 2:**
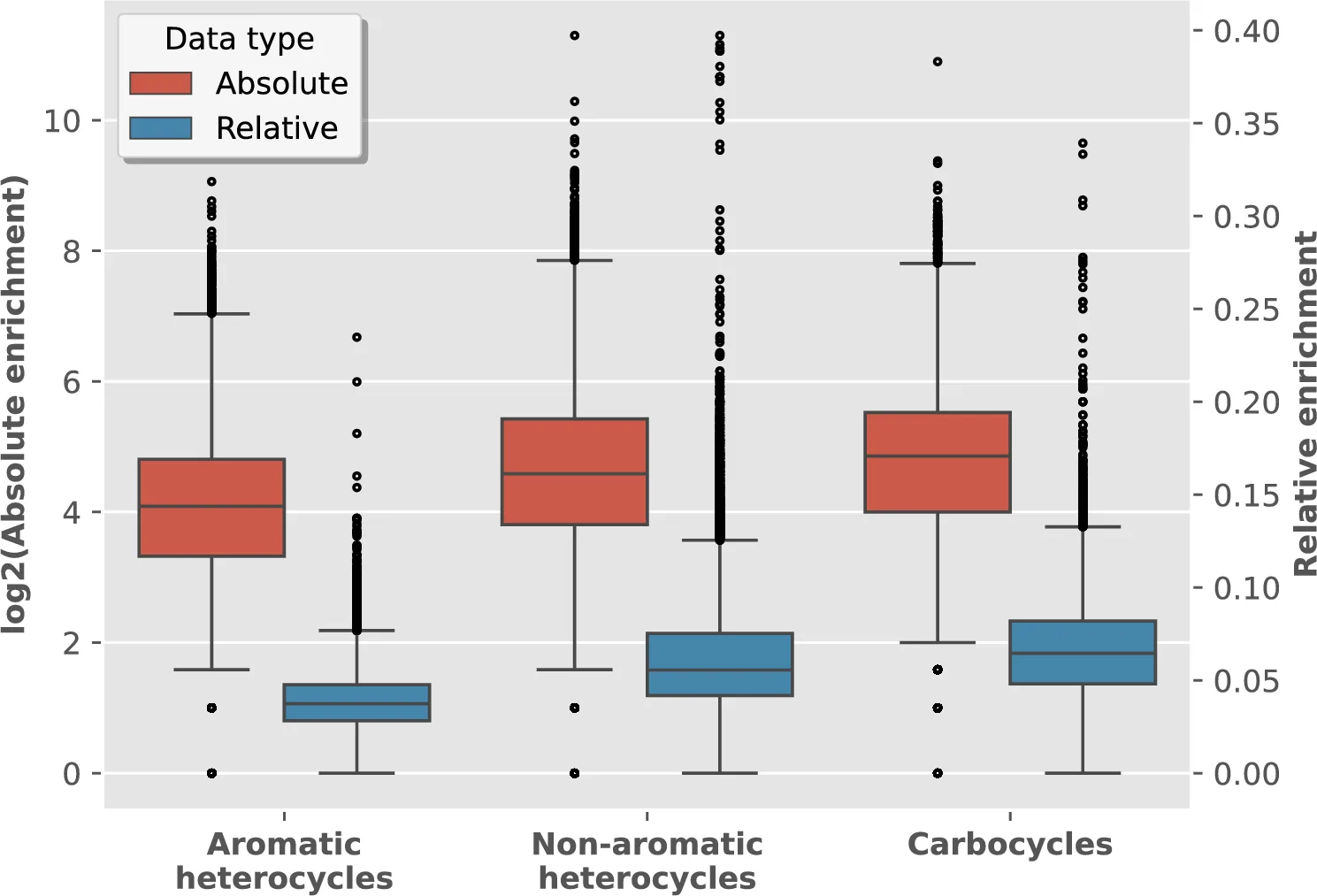
Distribution of absolute (red, left side) and relative (blue, right side) enrichment of aromatic heterocycles, non-aromatic heterocycles, and carbocycles in proteins

For the tissue and organ level, expression values from the HPA and PtDB databases were included in the analysis. The HPA database consists of two datasets, transcriptomics data that provide a snapshot of gene expression in an adult human, and proteomics data that quantify protein levels using specific antibodies via immunohistochemistry. However, the HPA proteomics dataset does not contain any numerical values; it only contains expression levels labelled ‘Not detected’, ‘Low’, ‘Medium’, and ‘High’. To simplify things and enable comparison with other datasets, the labels were mapped to the values ‘0’, ‘1’, ‘2’, and ‘3’, in that order. The last dataset is proteomics data from the PtDB, which quantifies protein abundance using mass spectrometry. Proteins with an expression value of zero across all tissues and organs were discarded from the respective datasets. It is important to note that expression data were not available for each protein in all three databases.

The tissue and organ classifications of the proteins were based on Terminologia Anatomica IDs (TA98), which comprised 57 tissues and 16 organs. These IDs are sequences of numbers separated by dots, beginning with the letter ‘A’ (e.g., ‘A12.1.00.001’ for ‘heart muscle’). The first two digits denote an organ (e.g., ‘A12’ for ‘cardiovascular system’). In this way, each expression value can be uniquely assigned to a tissue or organ, thereby circumventing naming discrepancies when using data from different databases. For each protein, we multiplied the total number of amino acids and the number of selected residues by the protein’s expression value. These weighted values were then summed across all proteins within each tissue and organ to obtain the total amino acid and residue content. Next, we calculated the overall enrichment of selected amino acids in each tissue and organ by dividing the total content of selected residues by the total amino acid content. For tissues, 13 showed no expression in the HPA transcriptomic dataset (18,975 proteins), nine in the HPA proteomic dataset (10,845 proteins), and 18 in the PtDB proteomic dataset. For organs, five showed no expression in the two HPA datasets and four in the PtDB proteomics dataset (16,539 proteins). For the MS data, staining values were collected for 11 organs.

**Fig. 3:**
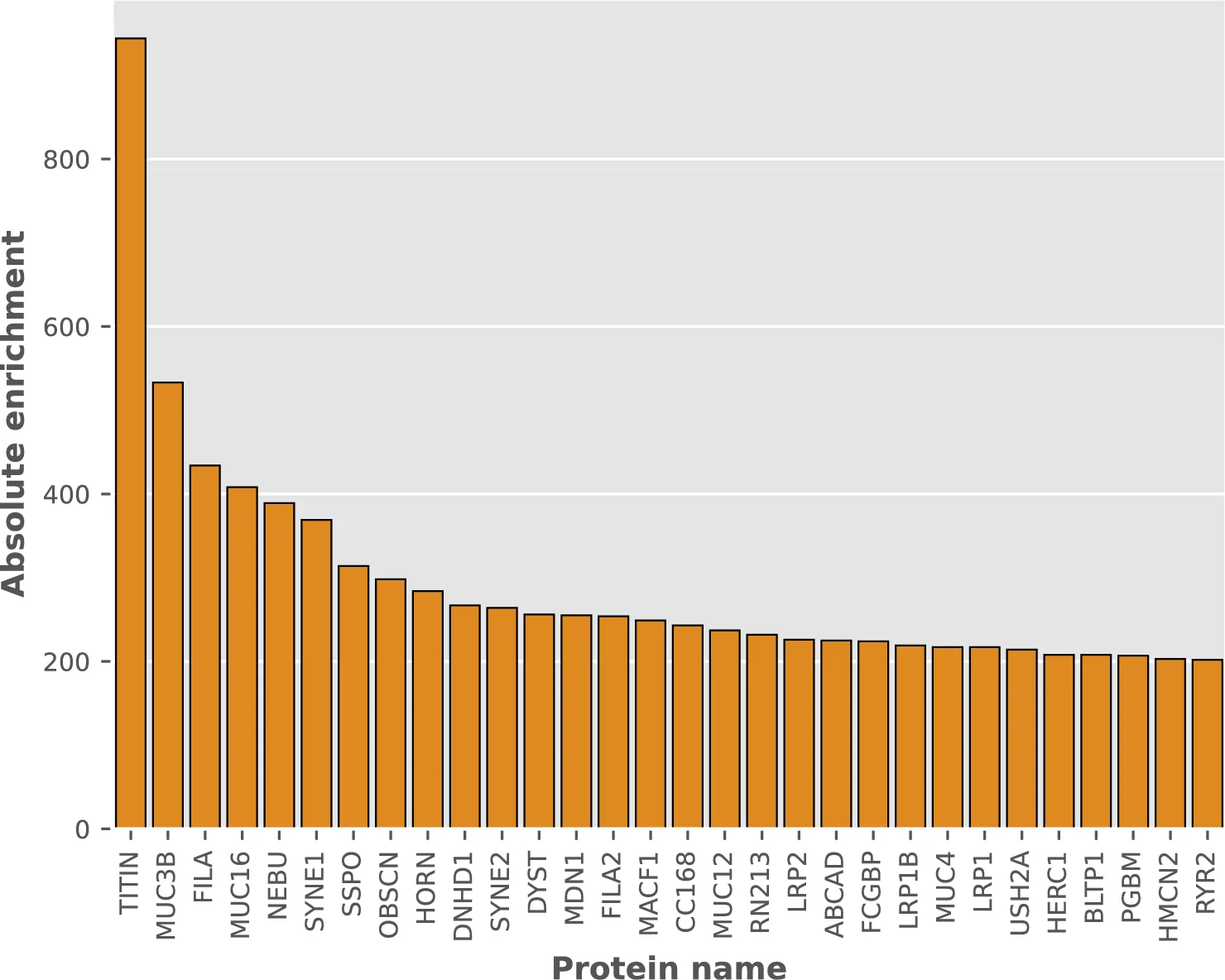
The top 30 proteins based on absolute aromatic heterocyclic enrichment

To identify proteins and families with an over-representation of these amino acid groups compared to the rest, we calculated the *Z*-score, which measures the number of standard deviations a value deviates from the mean (Huck et al. 1986). This means that a *Z*-score of 1 is one standard deviation from the mean and 3 is three standard deviations (typically defined as outliers), and so on. Proteins and families were ranked by the *Z*-scores of their absolute and relative values to emphasize those with particularly high proportions of aromatic heterocycles, non-aromatic heterocycles, carbocycles, and all other amino acids.

**Table 1:** The top ten human proteins with the absolute highest number of aromatic heterocycles, along with their corresponding *Z*-scores

Gene name	Full name	#Aromatic heterocycles	*Z*-score
TITIN	Titin	944	40.3
MUC3B	Mucin-3B	533	22.3
FILA	Filaggrin	434	18.0
MUC16	Mucin-16	408	16.9
NEBU	Nebulin	389	16.1
SYNE1	Nesprin-1	369	15.2
SSPO	SCO-spondin	314	12.8
OBSCN	Obscurin	298	12.1
HORN	Hornerin	284	11.5
DNHD1	Dynein heavy chain domain-containing protein 1	267	10.7

For each of the three expression datasets (regarding both tissues and organs), permutation-based *p* values for enrichment of aromatic heterocyclic residues were calculated. Multiple testing correction within each dataset was performed using the Benjamini–Hochberg false discovery rate procedure (*q* values). Tissues and organs with adjusted *q* values below 0.05 were considered statistically significant in their enrichment (Benjamini and Hochberg 1995). Additionally, the expression-weighted aromatic heterocyclic residue load was normalized by the number of expression entries at the tissue level and by the number of tissues and expression entries at the organ level.

Lastly, due to the low number of data points, Kendall rank correlation coefficient (τ) tests were applied to the tissue and organ datasets as well as the MS dataset to evaluate relationships within a dataset (e.g., protein count vs enrichment of selected residues) and relationships between datasets (e.g., enrichment of selected residues in organs vs intensity of iodine staining) (Kendall 1938).

## Results

### Data overview and proteins

There were 436,602 aromatic heterocyclic residues overall and 21.5 on average (3.9%), 718,775 non-aromatic heterocycles overall and 35.4 on average (6.2%), 718,401 carbocycles overall and 35.3 on average (6.6%), and 9,509,854 other residues overall and 467.8 on average (93.3%) (Fig. 2). The overall amino acid amount was 11,383,632 residues with an average of 560.

**Fig. 4:**
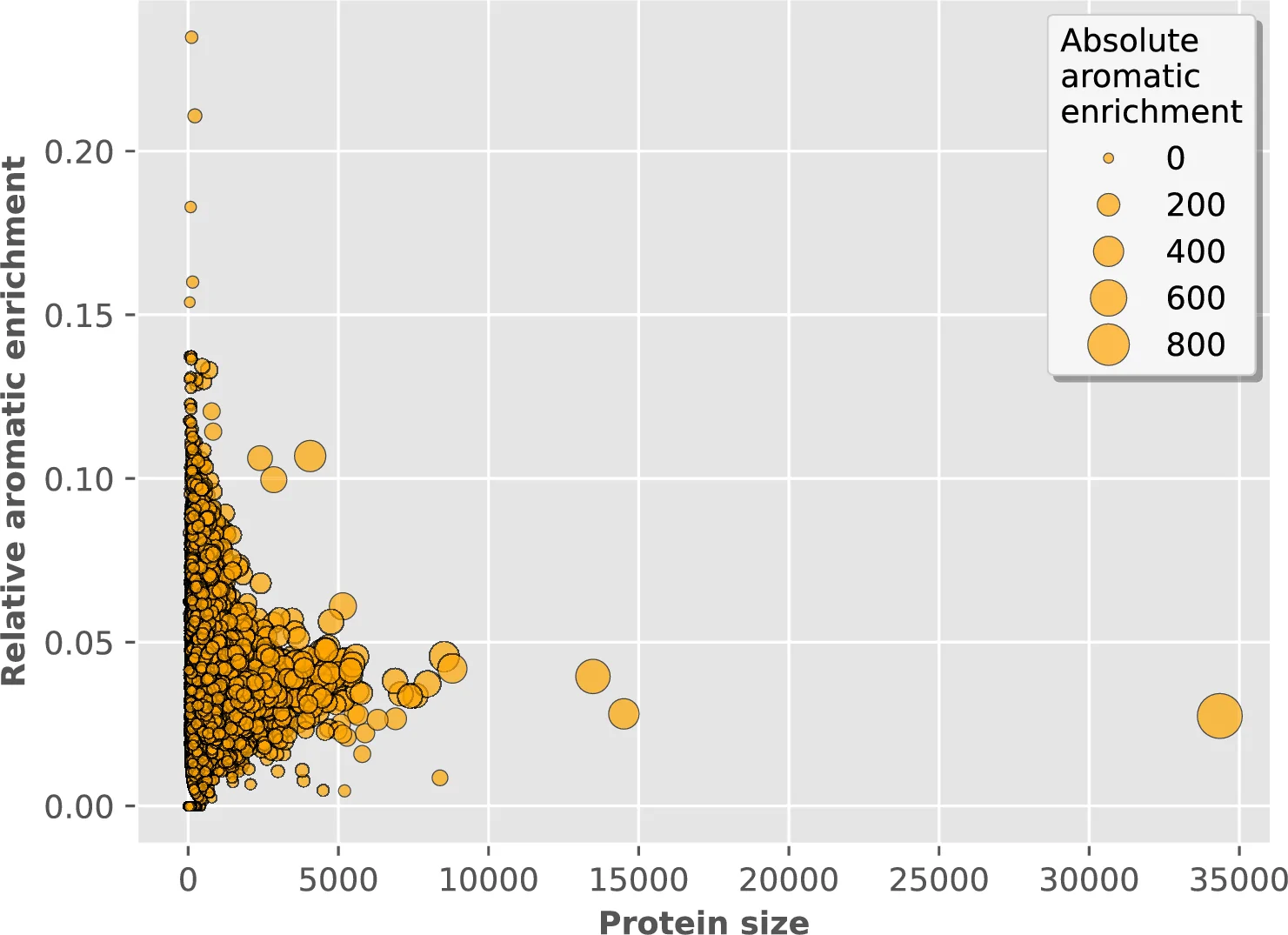
Relative aromatic enrichment of proteins against protein size. The size of each point corresponds to absolute aromatic enrichment. The lower-right outlier is the protein titin (*TTN*)

Among proteins with the highest aromatic heterocyclic amino acid content, titin (*TTN*) stands out with 944 occurrences in its 34,350-residue sequence (Z=40.3), followed by mucin-3B (*MUC3B*) with 533 in 13,477 residues (Z=22.34) and filaggrin (*FILA*) with 434 in 4061 residues (Z=18.0) (Fig. 3, Table 1). Naturally, larger proteins tend to have higher absolute counts simply because of their size, although their proportion of heterocyclic amino acids relative to total length remains low (Fig. 4). For example, only 2.7% of titin’s residues are heterocyclic, which is below the dataset-wide average of 3.9%. The proteins showed an absolute standard deviation of 22.9 residues for aromatic heterocycles.

**Fig. 5:**
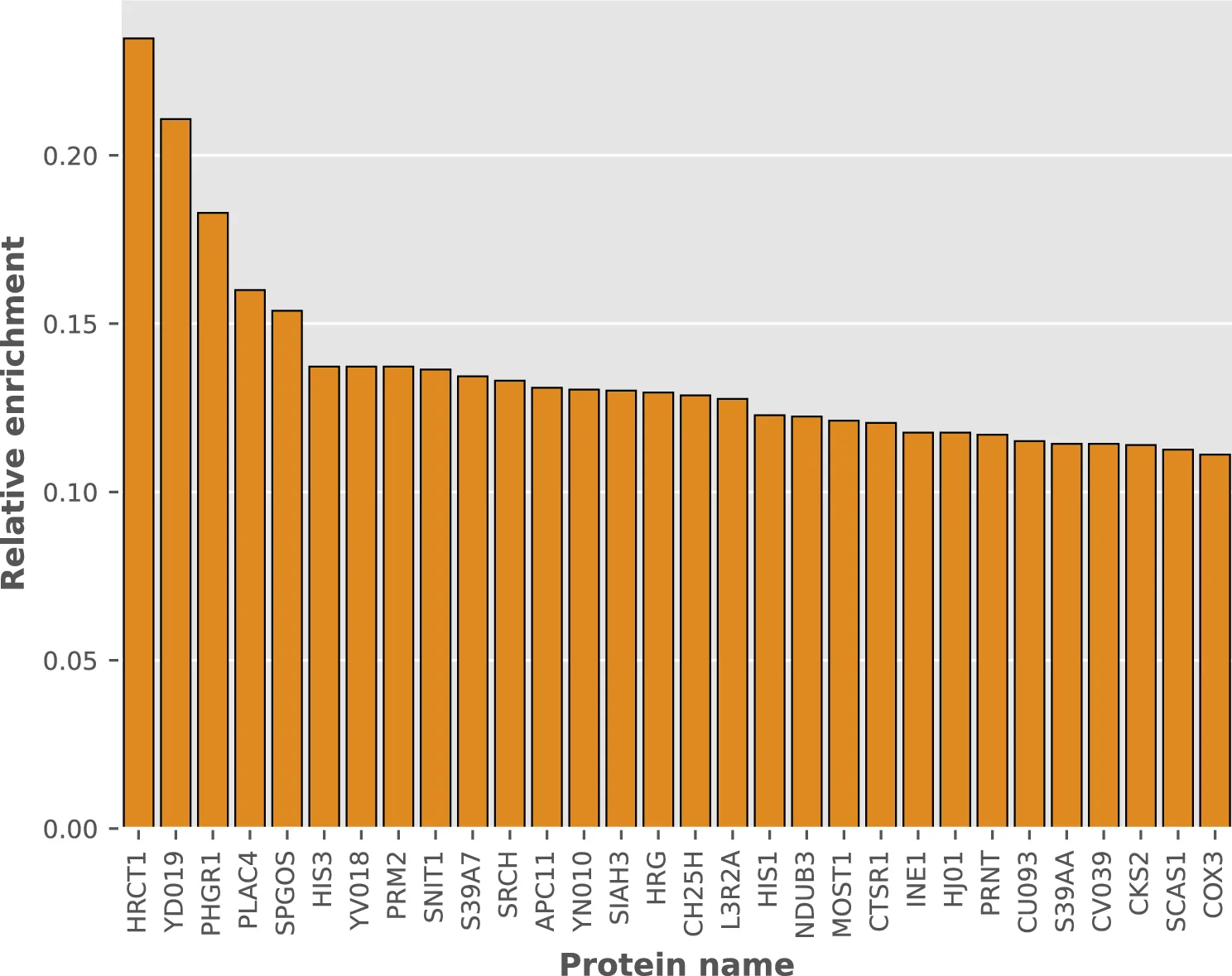
The top 30 proteins based on relative aromatic heterocyclic enrichment

**Table 2:** The top ten human proteins with the relatively highest number of aromatic heterocycles, along with their corresponding *Z*-scores

Gene name	Full name	%Aromatic heterocycles	*Z*-score
HRCT1	Histidine-rich carboxyl terminus protein 1	23.5	11.79
YD019	Putative uncharacterized protein FLJ46204	21.1	10.34
PHGR1	Proline, histidine, and glycine-rich protein 1	18.3	8.67
PLAC4	Placenta-specific protein 4	16.0	7.29
SPGOS	Putative uncharacterized protein SPART-AS1	15.4	6.92
PRM2	Protamine-2	13.7	5.92
HIS3	Histatin-3	13.7	5.92
YV018	Putative uncharacterized protein DKFZp434K191	13.7	5.92
SNIT1	Uncharacterized protein encoded by SND1-IT1	13.6	5.86
S39A7	Zinctransporter SLC39A7	13.4	5.74

In contrast, among proteins with an enrichment of heterocyclic amino acid content exceeding 10%, histidine-rich carboxyl terminus protein 1 (*HRCT1*) has the highest proportion, with 23.4% of its 115 residues being aromatic heterocycles (Z=11.8) (Fig. 5, Table 2). It is followed by putative uncharacterized protein FLJ46204 (*YD019*) and proline, histidine, and glycine-rich protein 1 (*PHGR1*), with 18.3% (Z=10.3) and 16.0% (Z=8.7) in sequences of 223 and 82 residues, respectively. Since even a few occurrences significantly impact small proteins, they are more likely to exhibit high relative proportions despite their low absolute counts (Fig. 4). For example, the *PHGR1* protein contains only 15 aromatic heterocyclic amino acids, below the dataset-wide average of 21.5 residues. The proteins showed a relative standard deviation of 1.7% residues for aromatic heterocycles.

### Families

**Fig. 6:**
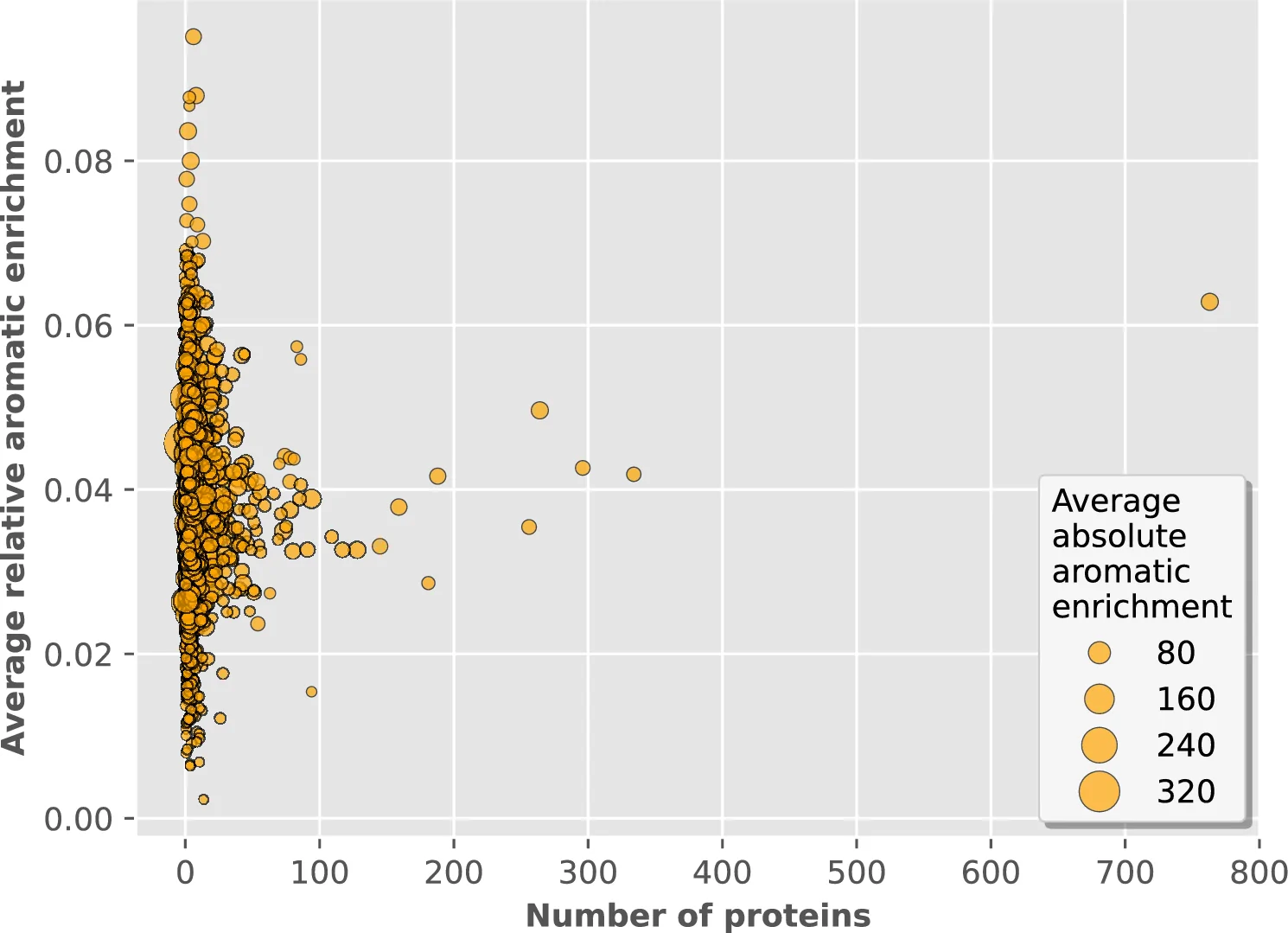
Average relative aromatic enrichment of protein families against protein family size. The size of each point corresponds to average absolute aromatic enrichment. The upper right outlier is the zinc fingers C2H2-type family

In contrast to the results of the proteins, no relationship between family size and either absolute or relative enrichment of aromatic heterocycles can be seen (Fig. 6).

**Fig. 7:**
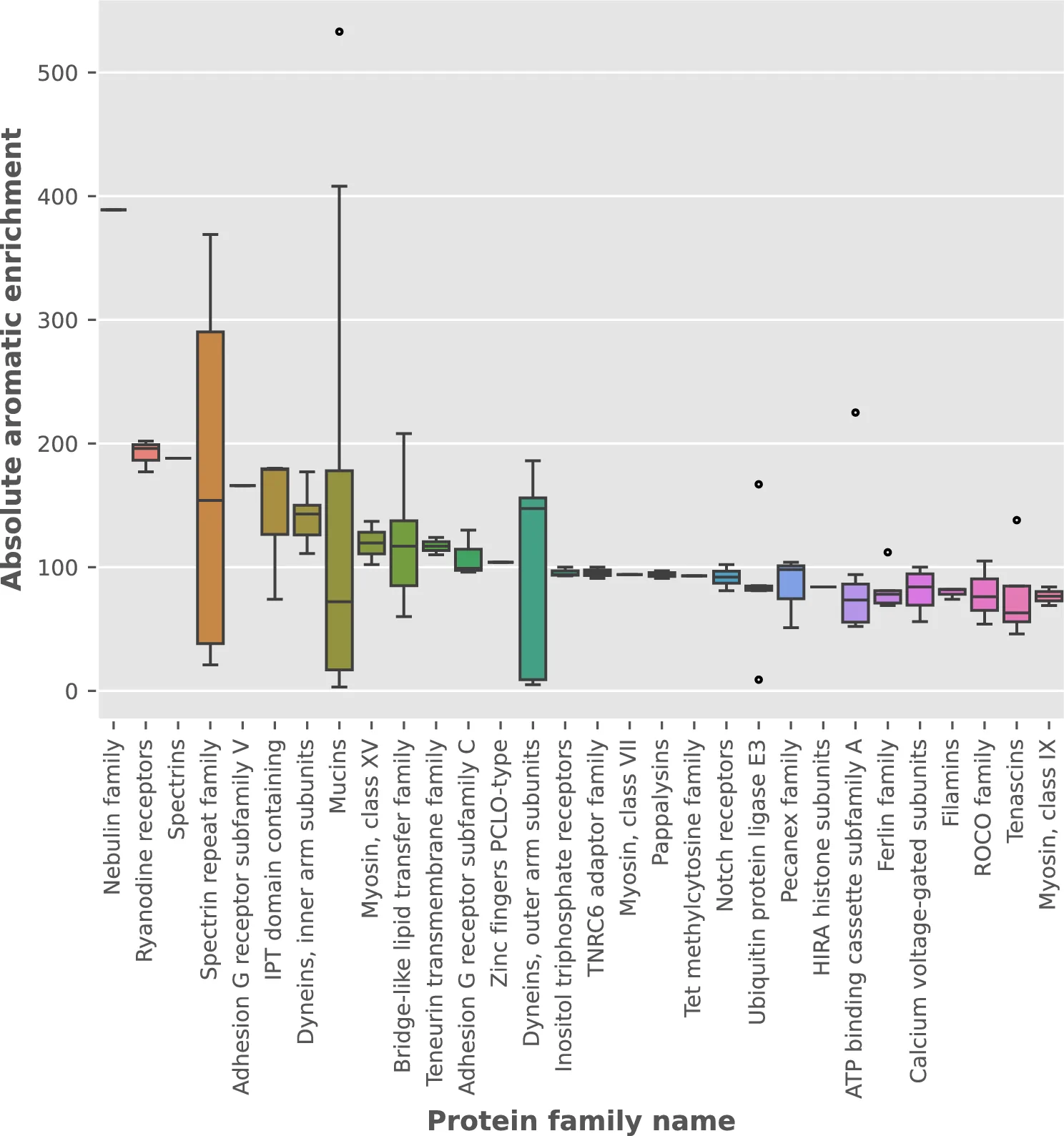
The top 30 protein families based on average absolute aromatic heterocyclic enrichment. Some names have been shortened for better visualization

The top families in terms of absolute aromatic enrichment are primarily protein families comprising large proteins but in small quantities (Fig. 7, Table 3). The family with the highest average number of aromatic heterocycles is the nebulin family with 389 heterocyclic residues (Z=17.5) and one protein. This is followed by the ryanodine receptor family with an average of 191.7 aromatic heterocyclic residues (Z=8) given three proteins and the spectrin family with an average of 188 aromatic heterocyclic residues (Z=7.9) given one protein. Overall, the results regarding the absolute enrichment of aromatic residues show that protein families are driven more by long, scaffold-like, structural or receptor proteins than by the quantity of proteins. The families showed an absolute standard deviation of 20.97 residues for aromatic heterocycles.

**Table 3:** The top ten protein families with the average absolute highest number of aromatic heterocycles, along with the number of proteins and their corresponding *Z*-scores

Gene family name	#Proteins	#Aromatic heterocycles	*Z*-score
Nebulin family	1	389	17.5
Ryanodine receptors	3	191.7	8
Spectrins	1	188	7.9
Spectrin repeat family	4	174.5	7.2
Adhesion G receptor subfamily V	1	166	6.8
IPT domain-containing	3	144.3	5.8
Dyneins, inner arm subunits	7	140.4	5.6
Mucins	19	120.1	4.6
Myosin, class XV	2	119.5	4.6
Bridge-like lipid transfer family	7	118.6	4.6

Some names have been shortened

**Fig. 8:**
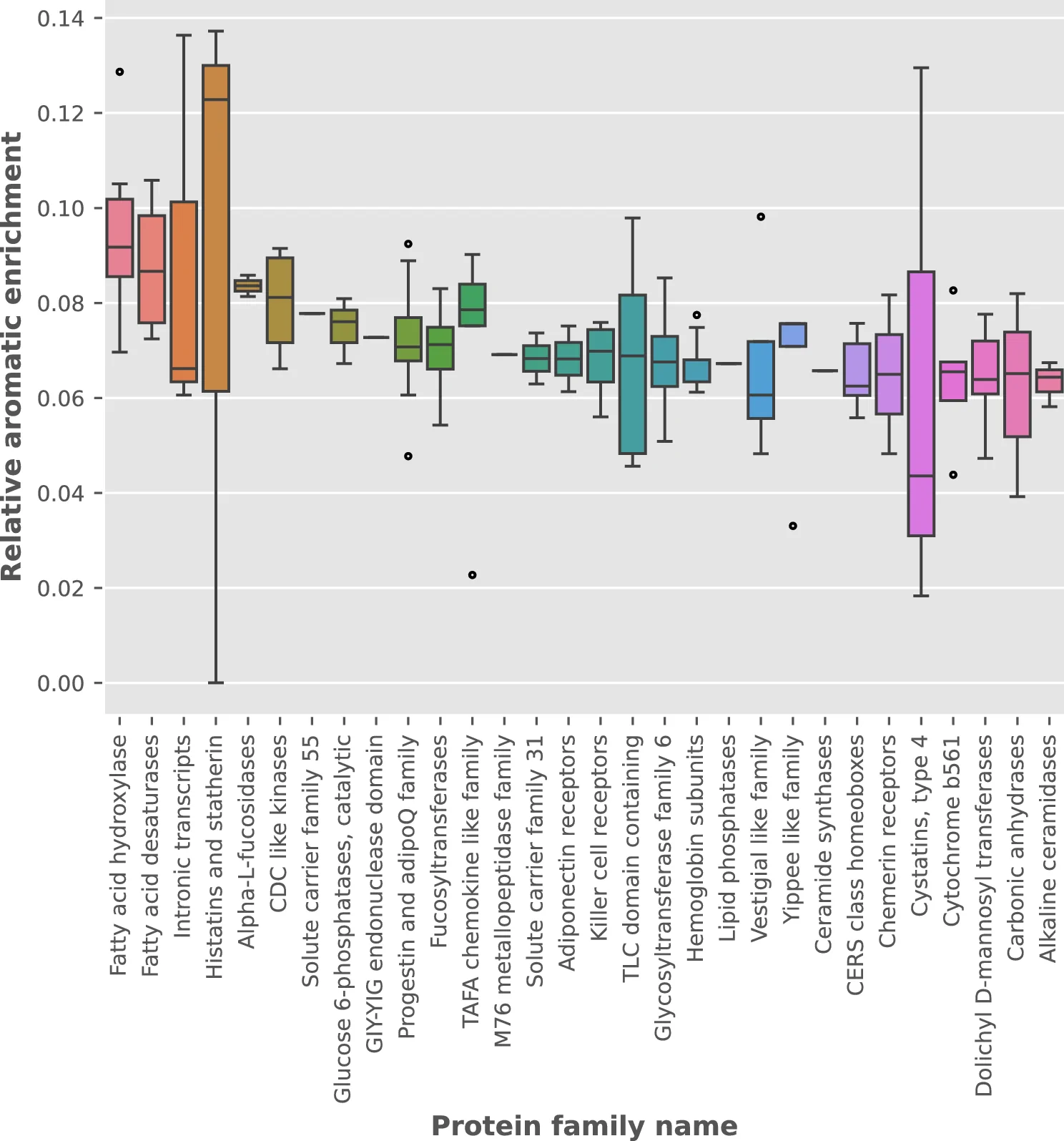
The top 30 protein families based on average relative aromatic heterocyclic enrichment. Some names have been shortened for better visualization

Protein families with high enrichment of aromatic heterocycles are dominated by enzymes responsible for membrane and lipid metabolism. As with the absolute results, these families largely have low protein content in common (<10) (Fig. 8, Table 4). The highest enrichment is found in the family of fatty acid hydroxylase domain-containing proteins, with 9.5% (Z=5) and six proteins, followed by fatty acid desaturases with 8.8% (Z=4.4) and eight proteins. In third place is the family of intronic transcripts with equal enrichment of 8.8% (Z=4.4) but two proteins. Regarding the top protein families of the absolute results, these rank much lower in comparison, namely a distribution across the entire dataset (Table 5). For example, the highest rank of those families at 164 is the spectrin family with average relative enrichment of 5.1% (Z=1.2). At the bottom is the mucin family at rank 1202 with average relative enrichment of 2.8% given 19 proteins (Z=-0.9). The families showed a relative standard deviation of 1.14% residues for aromatic heterocycles.

**Table 4:** The top ten protein families with the average relative highest number of aromatic heterocycles, along with the number of proteins and their corresponding *Z*-scores

Gene family name	#Proteins	%Aromatic heterocycles	*Z*-score
Fatty acid hydroxylase	6	9.5	5
Fatty acid desaturases	8	8.8	4.4
Intronic transcripts	3	8.8	4.4
Histatins and statherin	3	8.7	4.3
Alpha-L-fucosidases	2	8.3	4
CDC like kinases	4	8	3.7
Solute carrier family 55	1	7.8	3.5
Glucose 6-phosphatases, catalytic	3	7.5	3.2
GIY-YIG endonuclease domain	1	7.3	3.1
Progestin and adipoQ family	9	7.2	3

Some names have been shortened

**Table 5:** Average relative aromatic enrichment, *Z*-scores, and ranks of the top protein families based on the average absolute aromatic enrichment

Gene family name	#Proteins	%Aromatic heterocycles	*Z*-score	Rank
Nebulin family	1	4.6	0.7	313
Ryanodine receptors	3	3.9	0.07	626
Spectrins	1	5.1	1.2	164
Spectrin repeat family	4	4.4	0.6	358
Adhesion G receptor subfamily V	1	2.6	− 1	1260
IPT domain-containing	3	4.4	0.5	394
Dyneins, inner arm subunits	7	3.5	− 0.2	842
Mucins	19	2.8	− 0.9	1202
Myosin, class XV	2	3.6	− 0.2	794
Bridge-like lipid transfer family	7	4.1	0.2	530

Some names have been shortened

### Tissues

**Fig. 9:**
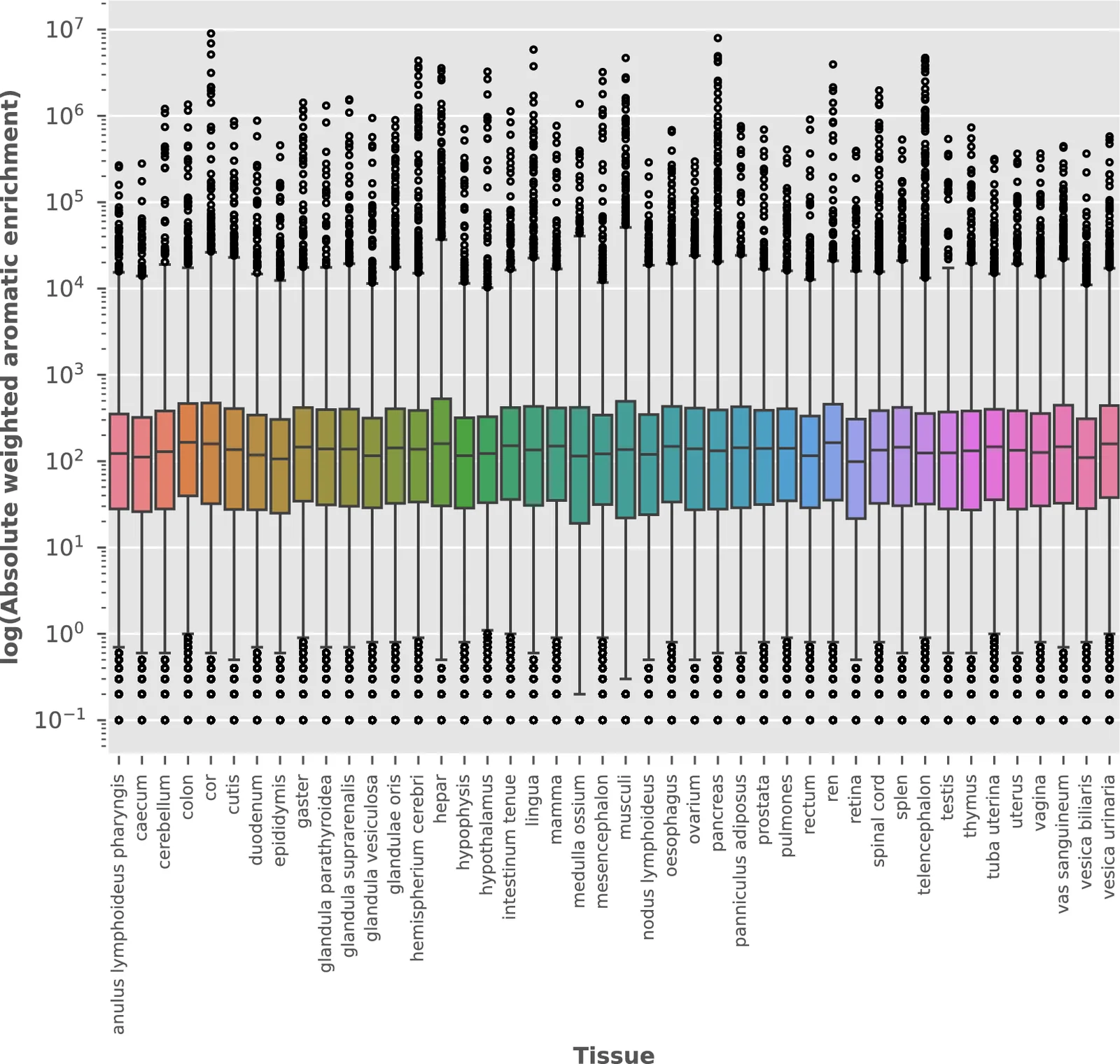
Logarithmic distribution of absolute weighted enrichment of aromatic heterocycles in tissues based on the expression values of the HPA transcriptomic dataset

**Fig. 10:**
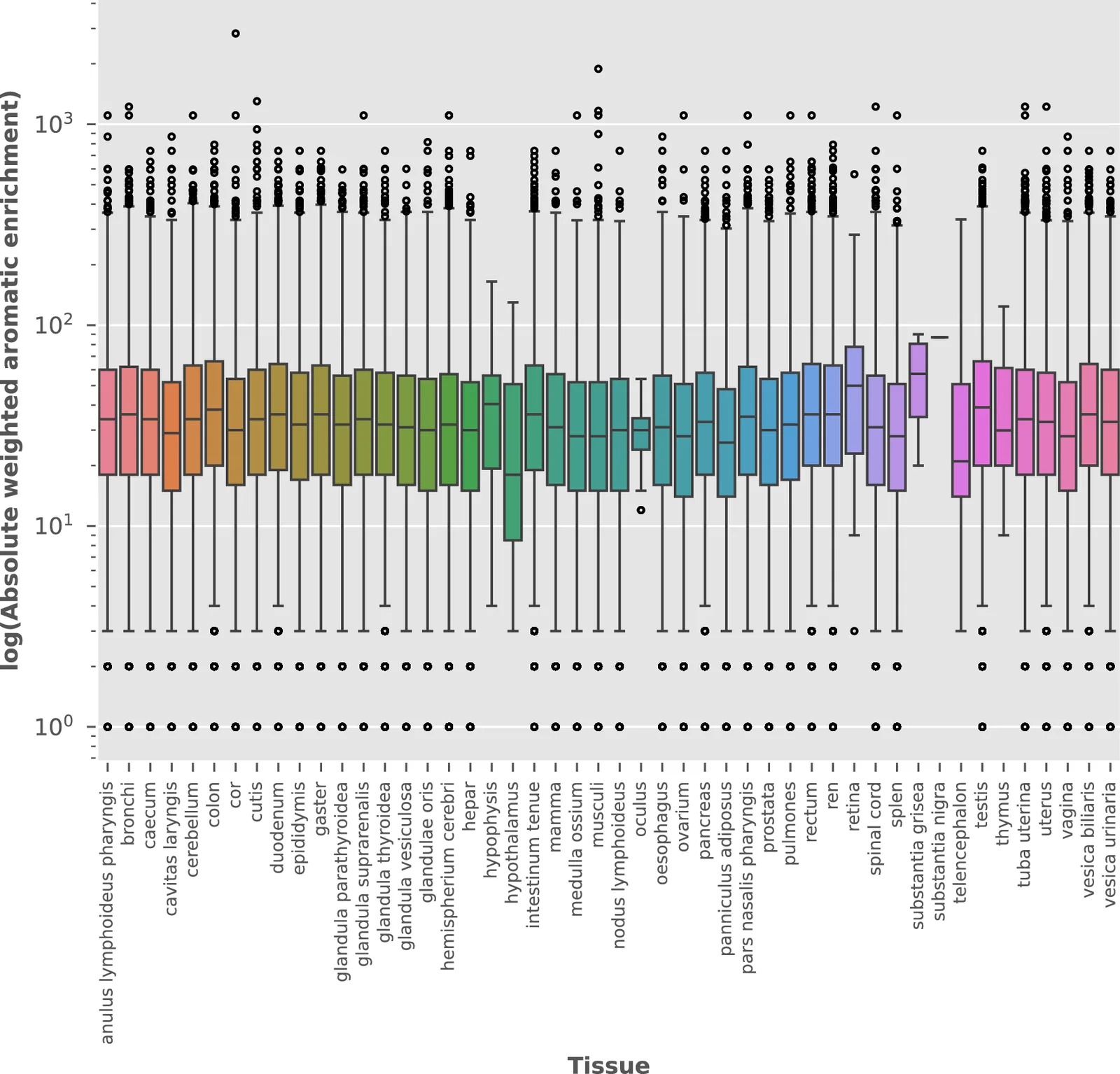
Logarithmic distribution of absolute weighted enrichment of aromatic heterocycles in tissues based on the expression values of the HPA proteomic dataset

**Fig. 11:**
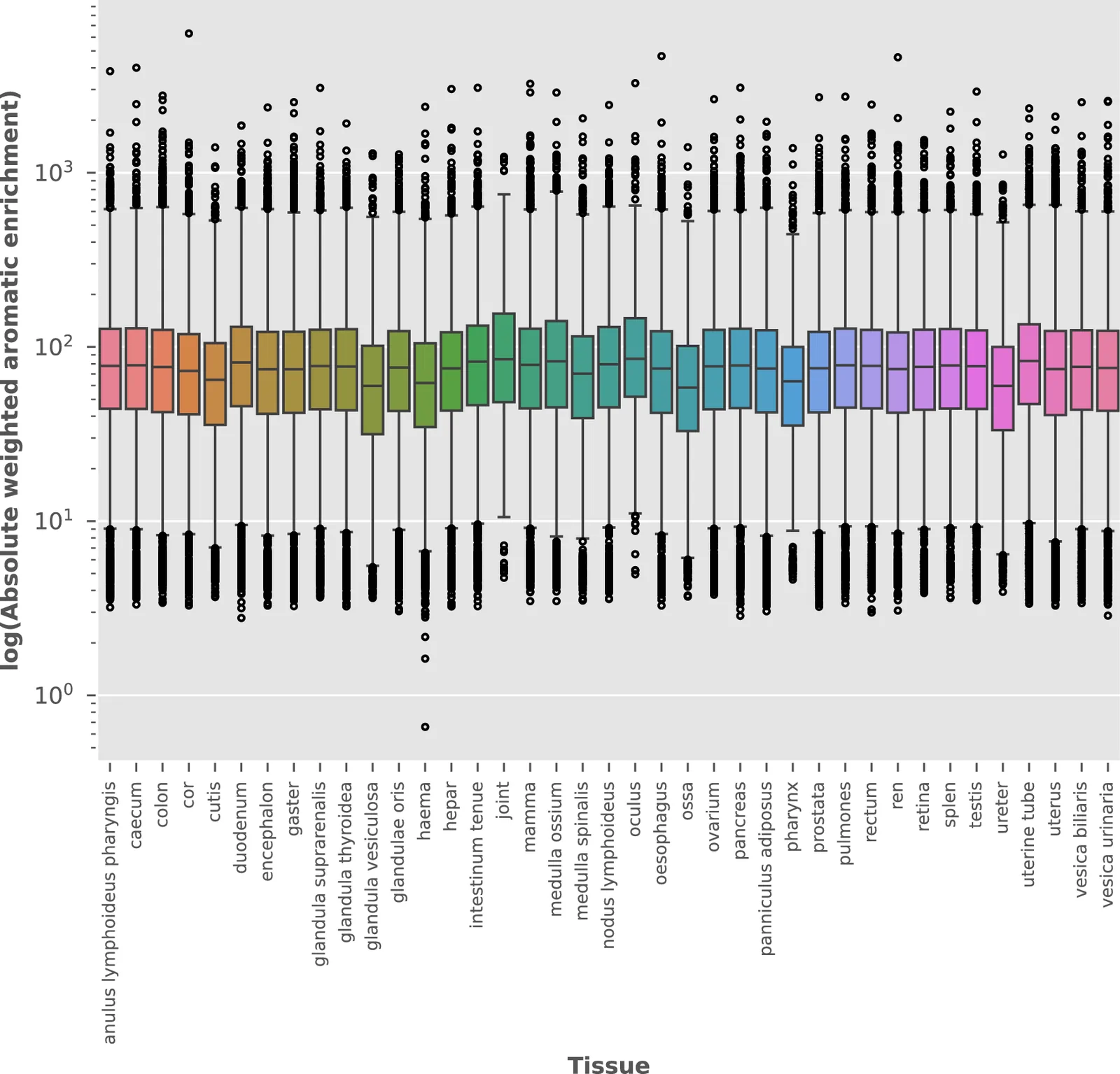
Logarithmic distribution of absolute weighted enrichment of aromatic heterocycles in tissues based on the expression values of the PtDB proteomic dataset

Across the three datasets (HPA transcriptomic, HPA proteomic, and PtDB proteomic), enrichment of aromatic heterocycles was highly similar, with little variation between them (Figs. 9, 10, and 11). Mean enrichment was 3.99% with a standard deviation of 0.39% in the HPA transcriptomic dataset, 3.63% with a standard deviation of 0.21% in the HPA proteomic dataset, and 3.54% with a standard deviation of 0.11% in the PtDB proteomic dataset. Furthermore, after *p* value correction, no tissue showed significant enrichment of aromatic heterocycles (q>0.05).

**Fig. 12:**
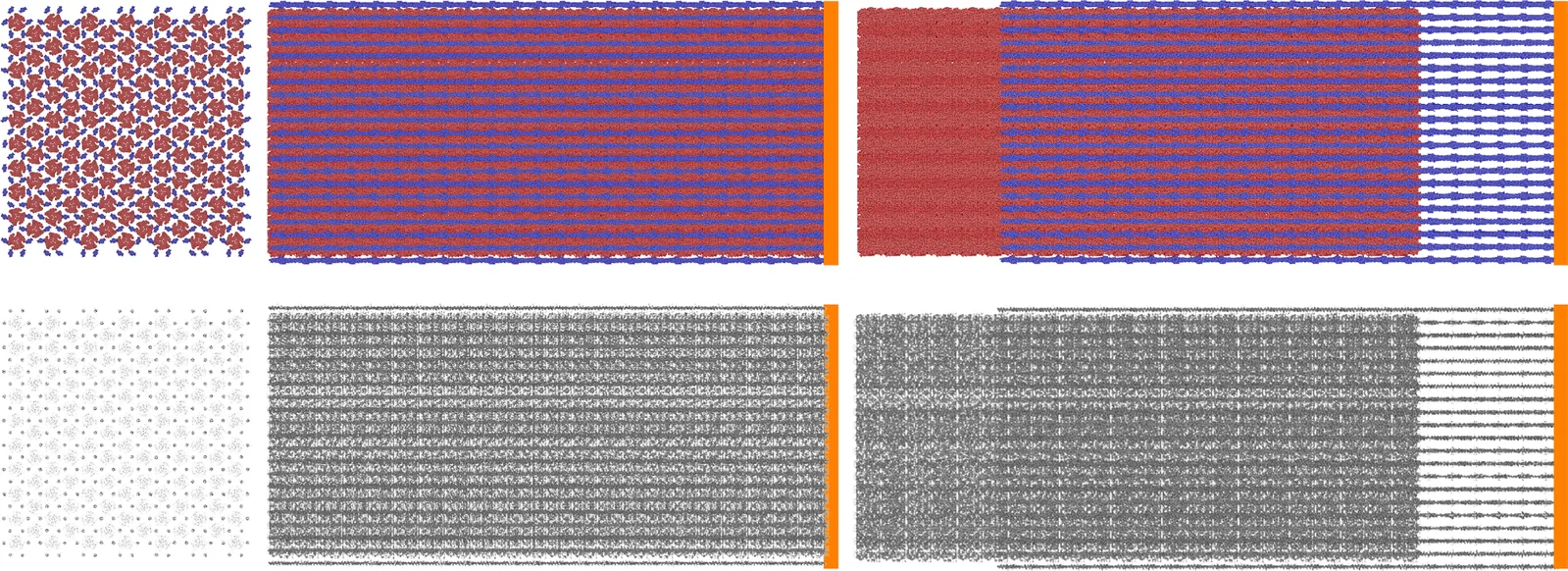
Half-sarcomere protein arrangements of the cardiac muscle (thin filaments with 7056 protein complexes PDB:6KN7 with a total of 54,902,736 AAs, and thick filaments with 2728 protein complexes PDB:8G4L with a total of 140,322,864 AAs) (Shiels and White 2008; Burbaum et al. 2021; Yamada et al. 2020; Dutta et al. 2023). From left to right: Cross-section at the level of the Z-line (orange), 100% overlap, and 75% overlap of the myosin (red) and actin filament (blue). Below, only the aromatic heterocycles are highlighted in black

**Fig. 13:**
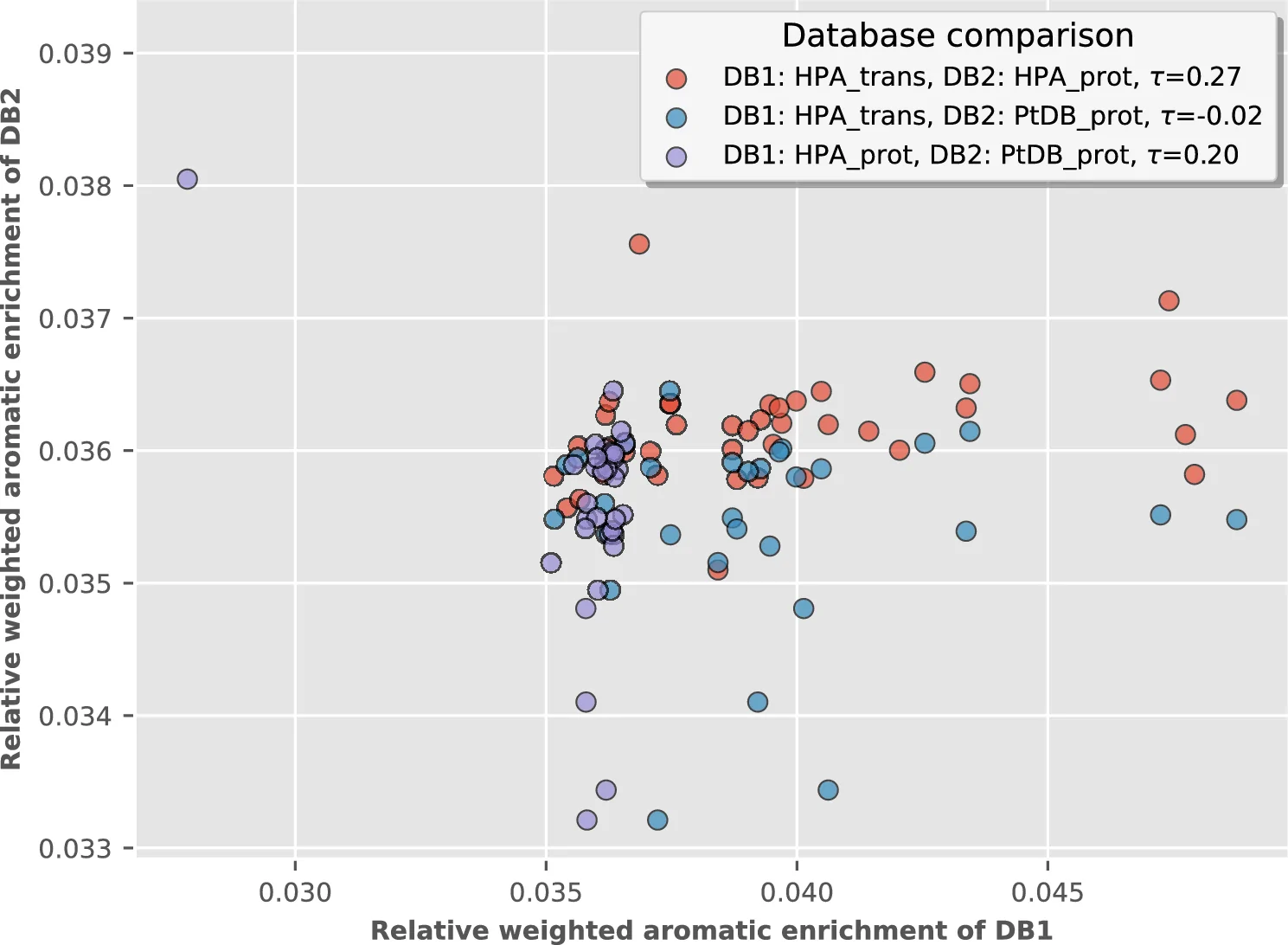
Aromatic heterocyclic enrichment in tissues between the HPA transcriptomic, HPA proteomic, and PtDB proteomic datasets. The *x*-axis shows the enrichment of the tissue based on the expression values of the first database (DB1), and the *y*-axis shows the enrichment of the tissue based on the expression values of the second database (DB2). Kendall rank correlation coefficients are indicated in the legend. Red, HPA transcriptomic vs HPA proteomic; blue, HPA transcriptomic vs PtDB proteomic; purple, HPA proteomic vs PtDB proteomic. The upper left outlier is the enrichment of the tissue *oculus* in the HPA and PtDB proteomic datasets

Furthermore, the top tissues differed for each dataset and showed no overlap. The top three tissues in the HPA transcriptomic dataset were heart (cor) (4.9%), telencephalon (4.8%), and hemispherium cerebrum (4.8%). The top three tissues in the HPA proteomic dataset were substantia nigra (4.7%), hypophysis (3.9%), and thymus (3.8%). Lastly, the top three tissues in the PtDB proteomic dataset were oculus (3.8%), pulmones (3.6%), and joint (3.6%). Additionally, the expression-weighted aromatic heterocyclic residue load was normalized by the number of expression entries at the tissue level and, separately, by the number of constituent tissues and expression entries at the organ level.

A comparison of the correlations of aromatic heterocyclic enrichment in tissues among the three expression databases showed weakly positive coefficients for the two HPA datasets (τ=0.27) and the two proteomic datasets (τ=0.2) (Fig. 13). This correlation was absent between the HPA transcriptomic and PtDB proteomic datasets (τ=-0.02).

Lastly, correlations between enrichment of aromatic heterocycles and other tissue statistics (such as number of proteins or expression-weighted amino acid amount) within each dataset revealed overall low coefficients (Tables 6, 7, and 8). Only medium coefficients of 0.37 and 0.45 can be seen for the HPA transcriptomic dataset between the enrichment and the overall amino acid amount as well as the overall aromatic heterocyclic amount in each tissue, respectively.

### Organs

**Fig. 14:**
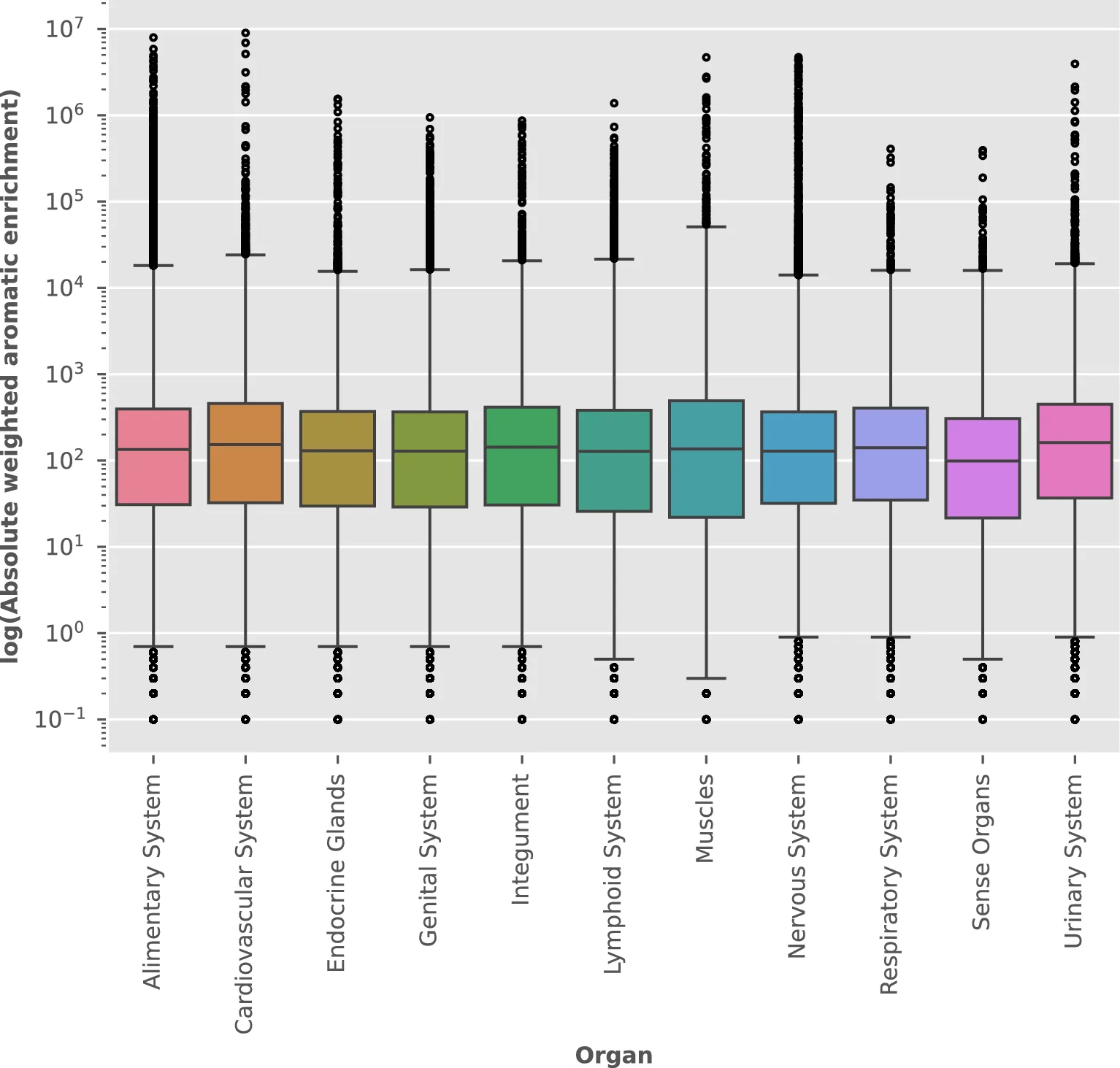
Logarithmic distribution of absolute weighted enrichment of aromatic heterocycles in organs based on the expression values of the HPA transcriptomic dataset

**Fig. 15:**
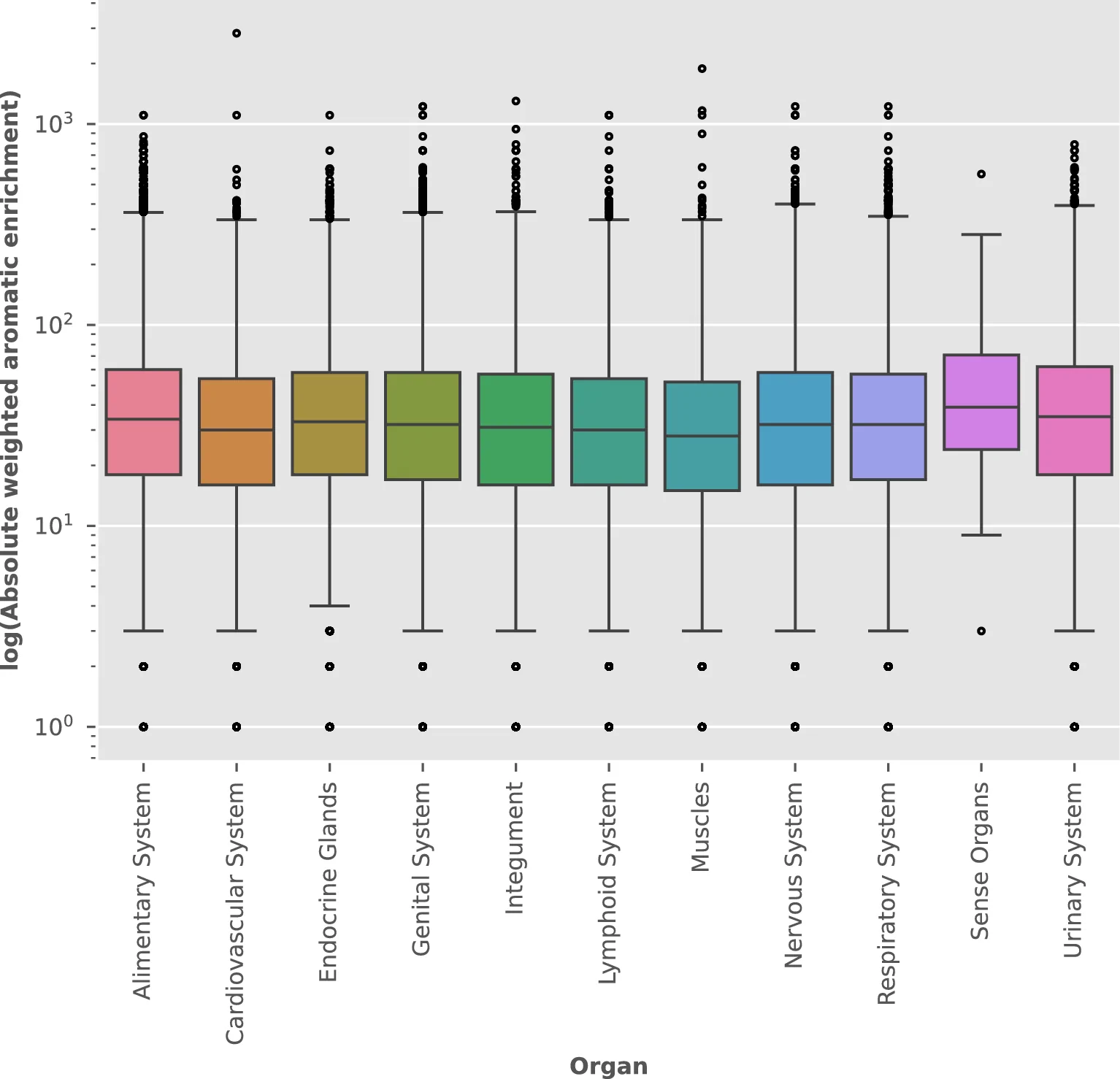
Logarithmic distribution of absolute weighted enrichment of aromatic heterocycles in organs based on the expression values of the HPA proteomic dataset

**Fig. 16:**
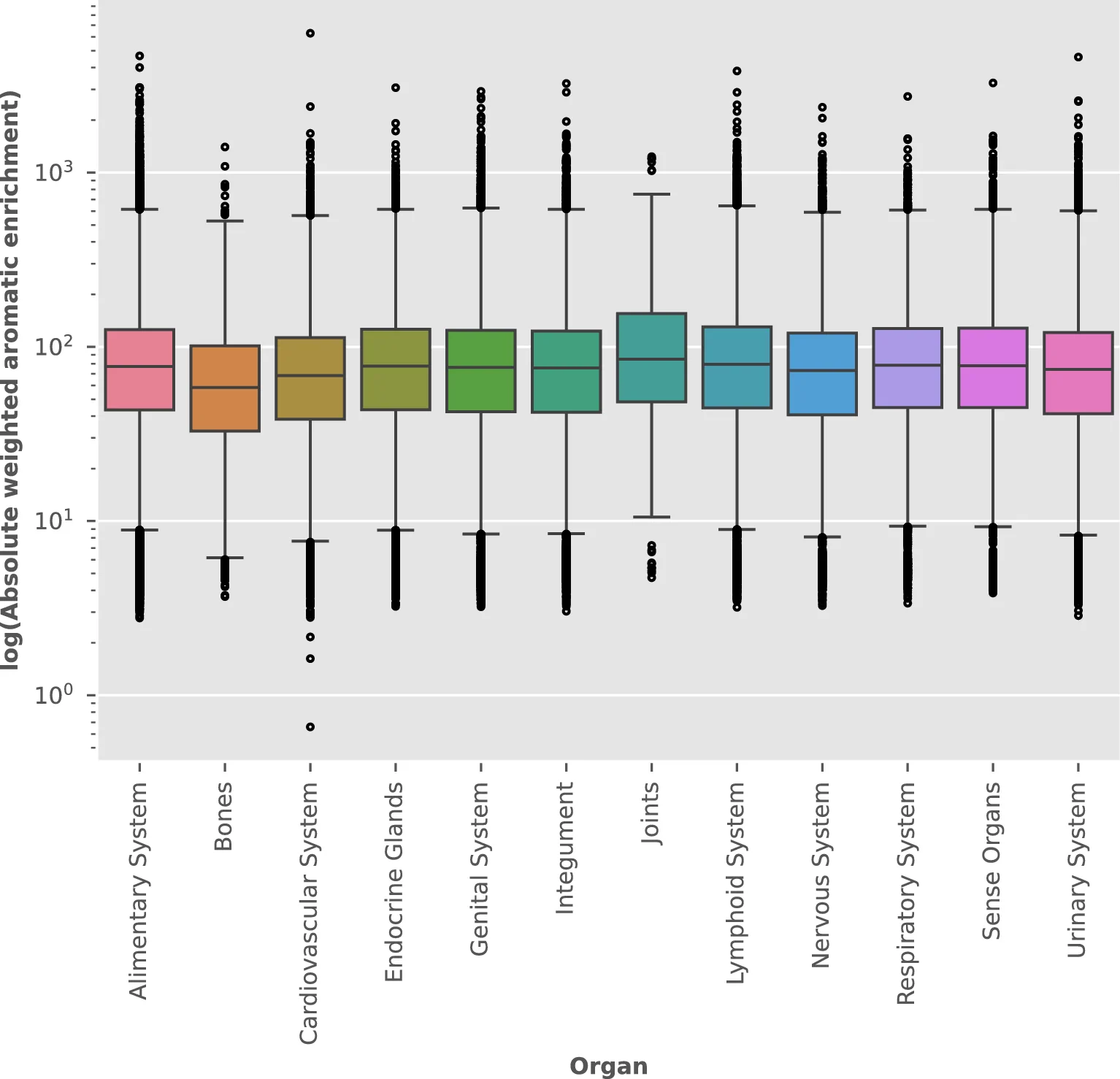
Logarithmic distribution of absolute weighted enrichment of aromatic heterocycles in organs based on the expression values of the PtDB proteomic dataset

As with the tissues, the variation in the enrichment of aromatic heterocycles in the organs was even lower across all three datasets (Figs. 14, 15, and 16). Mean enrichment values were 4.05% with a standard deviation of 0.32% in the HPA transcriptomic dataset, 3.6% with a standard deviation of 0.06% in the HPA proteomic dataset, and 3.55% with a standard deviation of 0.07% in the PtDB proteomic dataset. After *p* value correction, only the nervous system, the cardiovascular system, and the urinary system showed significant enrichment in the HPA transcriptomic dataset (q=0.039). This signal was not reproduced in either of the two proteomic datasets, or in any other tissue or organ in the transcriptomic dataset (q>0.05).

Looking at the top organs regarding enrichment, partial overlaps can be seen. The top three organs in the HPA transcriptomic dataset were the nervous system (4.6%), the cardiovascular system (4.5%), and the urinary system (4.3%). The top three organs in the HPA proteomic dataset were the cardiovascular system (3.6%), endocrine glands (3.6%), and urinary system (3.6%). Lastly, the top three organs in the PtDB proteomic dataset were the respiratory system (3.6%), joints (3.6%), and endocrine glands (3.6%).

**Fig. 17:**
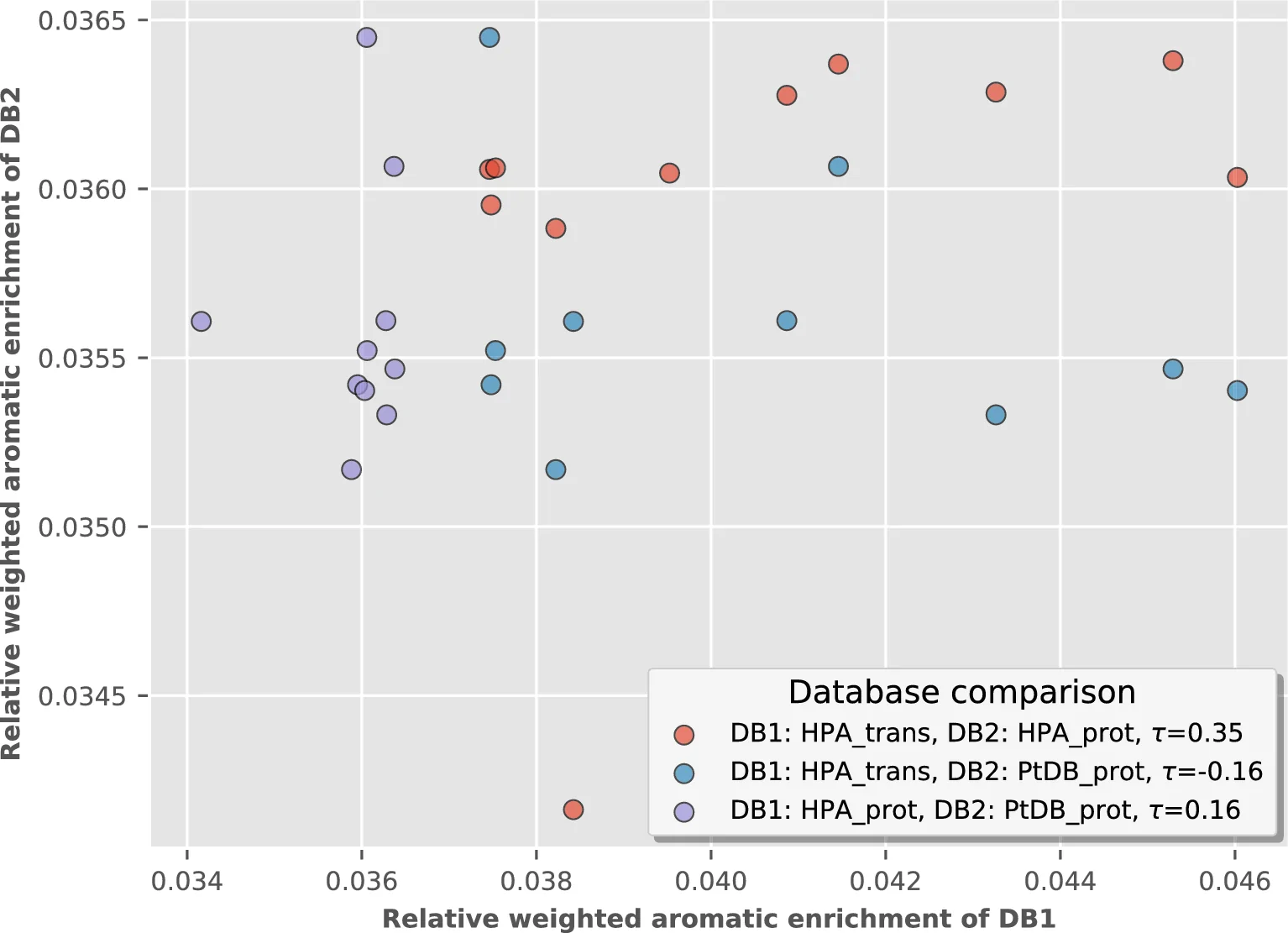
Aromatic heterocyclic enrichment in organs between the HPA transcriptomic, HPA proteomic, and PtDB proteomic datasets. The *x*-axis shows the enrichment of the organ based on the expression values of the first database (DB1), and the *y*-axis shows the enrichment of the organ based on the expression values of the second database (DB2). Kendall rank correlation coefficients are indicated in the legend. Red, HPA transcriptomic vs HPA proteomic; blue, HPA transcriptomic vs PtDB proteomic; purple, HPA proteomic vs PtDB proteomic

Correlations of aromatic heterocyclic enrichment in organs among the three expression databases revealed, again, weakly positive coefficients for the two HPA datasets (τ=0.35) and the two proteomic datasets (τ=0.16) (Fig. 17). This correlation was weakly negative between the HPA transcriptomic and PtDB proteomic datasets (τ=-0.16).

**Fig. 18:**
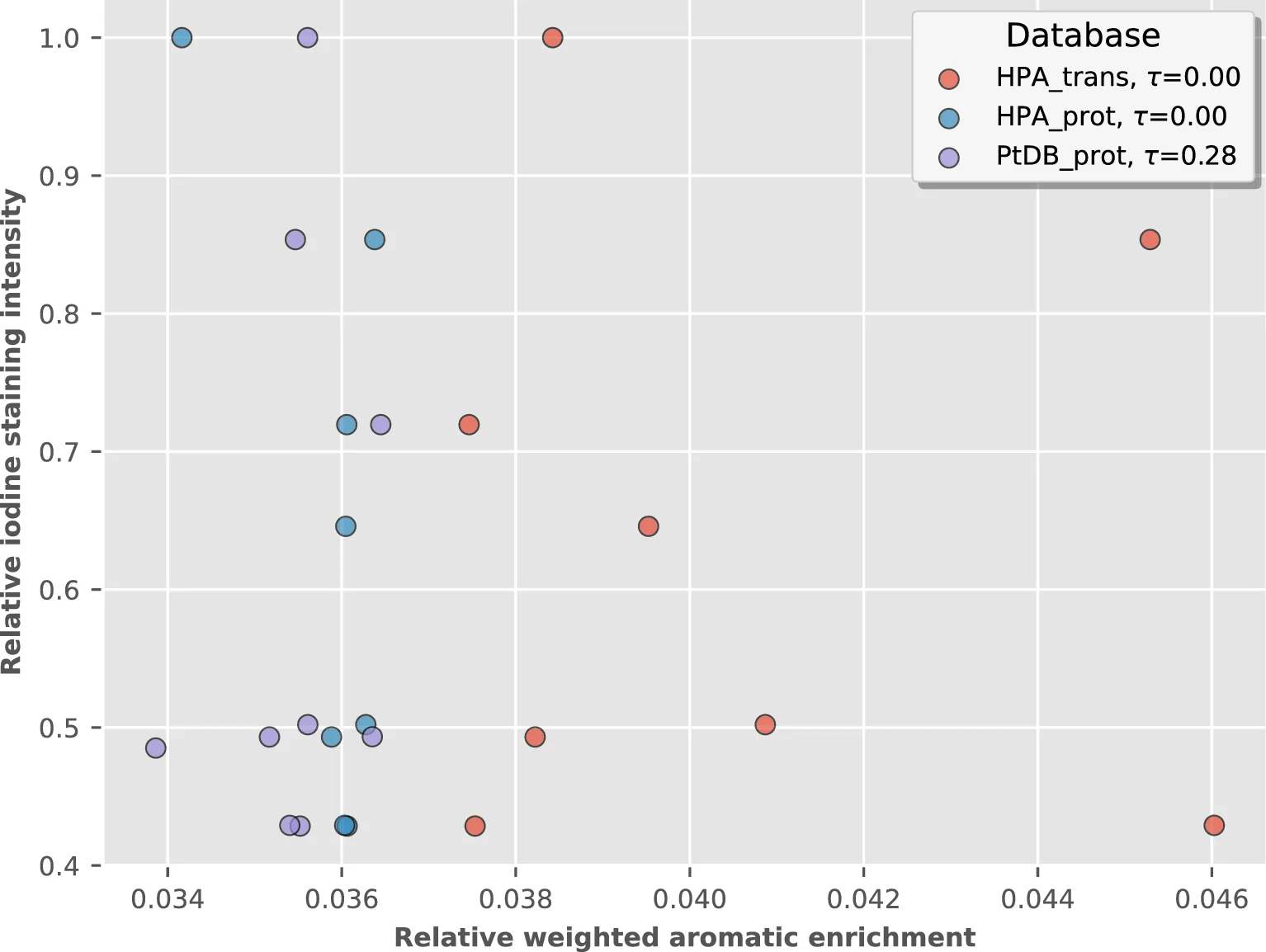
Aromatic heterocyclic enrichment in organs between the HPA transcriptomic, HPA proteomic, and PtDB proteomic datasets and relative iodine staining intensity. The *x*-axis shows the enrichment of the organ based on the expression values of the chosen database, and the *y*-axis shows the relative iodine staining intensity based on the values of the MorphoSource database. Kendall rank correlation coefficients are indicated in the legend. Red, HPA transcriptomic; blue, HPA proteomic; purple, PtDB proteomic

Lastly, correlations between enrichment of aromatic heterocycles and other organ statistics (such as number of proteins or expression-weighted amino acid amount) within each dataset revealed overall weak and inconsistent coefficients (Tables 9, 10, and 11). Furthermore, looking at the relative iodine staining intensity of each organ compared to the enrichment values, correlations were absent in the two HPA datasets (τ=0.0) and only weakly positive in the PtDB proteomic dataset (τ=0.28) (Fig. 18). Interestingly, weak to medium negative correlations were seen between the other chosen statistics and staining intensity.

## Discussion

### Proteins with numerous aromatic heterocycles

Of all proteins, titin contains the largest number of aromatic heterocycles. This is unsurprising, as it is the largest known human protein, with a total amino acid length of ∼ 30,000. Additionally, it is highly expressed in muscles, as it is a structural protein responsible for their tension (Freundt and Linke 2018). Other important structural proteins in the skeletal musculature are myosin (*MYH2*: 2.3%; *MYH4*: 2.5%; *MYH1*: 2.4%), actin (*ACTS*: 3.4%), nebulin (*NEBU*: 4.6%), and obscurin (*OBSCN*: 3.7%) (Holmes 2007; Dominguez and Holmes 2011). Nevertheless, a proportional comparison of titin with these structural proteins shows that myosins are rich in aromatic heterocycles (*TTN*: 2.7%). On the other hand, special isoforms of myosin (*MYH6*: 2.3%; *MYH7*: 2.6%) and actin (*ACTC1*: 3.4%) are found in the heart muscle (Fig. 12). The high proportion of aromatic heterocycles in the structural proteins also explains why the muscles, and in particular the heart muscles, are well stained by iodine (Metscher 2009b; Jeffery et al. 2011; Kupczik et al. 2015; Dickinson et al. 2018; Nyakatura et al. 2019; Kolmann et al. 2023).

Another structural protein family is the collagens, which are rich in proline but not in aromatic heterocycles (Mayne and Brewton 1993; Ricard-Blum 2011; Wesp et al. 2024). Exceptions are collagen types XX (FACITs), XVIII (endostatin precursor), XXVI (other), VI (network-forming), and XIV (FACITs) (*COKA1*: 3.5%; *COIA1*: 3.2%; *COQA1*: 2.7%; *CO6A5*: 2.7%; *COEA1*: 2.6%). Almost all type VI collagens (network-forming), which occur in the extracellular matrix, show a high number of aromatic heterocycles (*CO6A1*–*CO6A6*: 1.6-2.7%). In contrast, the most frequently occurring collagen types I (fibrillar), IV (network-forming), VIII (network-forming), IX (FACITs), and X (network-forming) contain only a few aromatic heterocycles (*CO1A1*: 1.0%; *CO1A2*: 1.5%; *CO4A5*: 1.1%; *CO8A1*: 1.6%; *COAA1*: 1.8%; *CO8A2*: 1.4%; *CO9A2*: 1.2%), with the exception of *CO4A4* (2.5%). Interestingly, the collagen family occupies an intermediate position in the protein family list at rank 194 for absolute value (average of 36.1) and sits close to the bottom at rank 1376 for relative values (average of 2%). This could be explained by the fact that, although collagens are relatively large and therefore naturally contain a high number of aromatic heterocycles, these proteins are not enriched in those residues. Averaging over the entire family further reduces this influence. This might also explain why areas between the fascicles within the muscle are less intensely stained, allowing the fascicles to be differentiated.

However, proteins in muscle and connective tissues are not the only ones with high aromatic heterocyclic content; others include keratin-associated proteins (max. 6.8%), fillagrin (*FILA*: 10.7% and *FILA2*: 10.6%), and hornerin (*HORN*: 10.0%) (Henry et al. 2011). They thus represent a group of iodine-sensitive proteins in the skin. This could explain the high contrast of this tissue when stained with iodine (Metscher 2009a).

Directly downstream in the gastrointestinal tract, the mucins (*MUC1*: 4.7%; *MUC2*: 2.1%; *MUC3A*: 2.5%; *MUC3B*: 4.0%; *MUC4*: 4.0%; *MUC5A*: 2.8%; *MUC5B*: 3.5%; *MUC6*: 4.7%; *MUC7*: 3.2%) with their high number of aromatic heterocycles represent another important group. Mucins are structural components of mucus on mucous membranes, including the gastrointestinal tract. We know from Fennerty (1999) that the oesophagus can be easily stained with iodine/Lugol’s solution. This suggests that mucins play a significant role in this process. Furthermore, increased mucin production is observed in many types of cancer (Singh and Bandyopadhyay 2007; Singh et al. 2008). *PHGR1* (18.3%), another gene expressed in the gastrointestinal tract, tops the relative proportions of aromatic heterocycles.

**Table 6:** Kendall rank correlation coefficients of enrichment of aromatic heterocycles in tissues between selected statistics in the HPA transcriptomic dataset

HPA_trans Kendall’s τ	#Tissues	#Proteins	AA amount	Arom. amount	Enrichment
#Tissues	1				
#Proteins	0.42	1			
AA amount	0.34	0.27	1		
Arom. amount	0.33	0.26	0.92	1	
Enrichment	0.19	0.07	0.37	0.45	1

**Table 7:** Kendall rank correlation coefficients of enrichment of aromatic heterocycles in tissues between selected statistics in the HPA proteomic dataset

HPA_prot Kendall’s τ	#Tissues	#Proteins	AA amount	Arom. amount	Enrichment
#Tissues	1				
#Proteins	0.29	1			
AA amount	0.28	0.93	1		
Arom. amount	0.28	0.93	0.99	1	
Enrichment	−0.08	0.12	0.11	0.12	1

**Table 8:** Kendall rank correlation coefficients of enrichment of aromatic heterocycles in tissues between selected statistics in the PtDB proteomic dataset

PtDB_prot Kendall’s τ	#Tissues	#Proteins	AA amount	Arom. amount	Enrichment
#Tissues	1				
#Proteins	0.26	1			
AA amount	0.29	0.94	1		
Arom. amount	0.28	0.95	0.98	1	
Enrichment	− 0.26	0.26	0.24	0.26	1

The best-known reaction involving iodine occurs in the thyroid gland (Colledge et al. 2014). However, this is an enzymatic reaction and is based on the production of iodine-containing thyroid hormones, such as triiodothyronine (T3) and thyroxine or tetraiodothyronine (T4), and the peptide hormone calcitonin. The bound iodine levels are negligible (1.17 ng/dL), as these hormones occur at very low concentrations relative to those of the aromatic heterocycles.

### Families, tissues, and organs

To analyse the large amount of data, we grouped the proteins into four levels of complexity: individual proteins, protein families, tissues, and organs. Organs were especially interesting, as we could directly compare their enrichment of aromatic heterocycles to iodine staining intensity, which was not available for families and tissues.

As mentioned in the Results section, families containing very large proteins rank among the highest for absolute aromatic heterocyclic enrichment, including nebulin family, ryanodine receptors, spectrins, dynein-related families, and mucins. On the other hand, families with the highest relative enrichment are dominated by smaller enzyme and membrane-associated families, such as fatty acid hydroxylase domain-containing family, fatty acid desaturases, and alpha-L-fucosidases. Overall, families primarily reflect protein size within a family in terms of absolute values, whereas in terms of relative values, they tend to reflect enrichment at the sequence level. Furthermore, it is important to note that there are far fewer enzyme proteins than structural proteins in tissues (Morris et al. 2022).

Generally speaking, the higher the level of complexity (protein → family → tissue → organ), the less distinguishable the ingroups become, as shown by the low variability in their enrichment. This suggests that while the enrichment of aromatic heterocyclic amino acids in tissues and organs may still be biologically significant, its signal is diluted when averaged across larger anatomical groups. Looking at the enrichment of these amino acids, we were only able to detect significant signals in the transcriptomic dataset for three organs (urinary, cardiovascular, and nervous systems), which were not reproduced for any tissues or any other organs in the proteomic datasets. While we observe high staining intensity (0.85) in the cardiovascular system, the intensity is comparatively low (0.43) in the nervous system and nonexistent in the urinary system. Furthermore, transcriptome data do not necessarily reflect protein levels in tissues/organs, which makes comparisons difficult. Therefore, the current results do not support any distinction between tissues and organs based solely on the enrichment of aromatic heterocyclic compounds.

**Table 9:** Kendall rank correlation coefficients of enrichment of aromatic heterocycles in organs between selected statistics in the HPA transcriptomic dataset as well as iodine staining intensity

HPA_trans Kendall’s τ	#Tissues	#Proteins	AA amount	Arom. amount	Enrichment	Staining
#Tissues	1					
#Proteins	0.95	1				
AA amount	0.76	0.71	1			
Arom. amount	0.69	0.64	0.93	1		
Enrichment	0.11	0.02	0.16	0.24	1	
Staining	−0.49	−0.43	−0.43	−0.43	0	1

**Table 10:** Kendall rank correlation coefficients of enrichment of aromatic heterocycles in organs between selected statistics in the HPA proteomic dataset as well as iodine staining intensity

HPA_prot Kendall’s τ	#Tissues	#Proteins	AA amount	Arom. amount	Enrichment	Staining
#Tissues	1					
#Proteins	0.77	1				
AA amount	0.77	1	1			
Arom. amount	0.77	1	1	1		
Enrichment	−0.06	0.13	0.13	0.13	1	
Staining	−0.52	−0.57	−0.57	−0.57	0	1

**Table 11:** Kendall rank correlation coefficients of enrichment of aromatic heterocycles in organs between selected statistics in the PtDB proteomic dataset as well as iodine staining intensity

PtDB_prot Kendall’s τ	#Tissues	#Proteins	AA amount	Arom. amount	Enrichment	Staining
#Tissues	1					
#Proteins	0.8	1				
AA amount	0.8	1	1			
Arom. amount	0.8	1	1	1		
Enrichment	−0.2	−0.09	−0.09	−0.09	1	
Staining	−0.12	−0.22	−0.22	−0.22	0.28	1

Analysis of the raw aromatic heterocyclic loads did not reveal a clear cross-dataset pattern at the tissue level, although the colon and gaster (stomach) ranked consistently among the strongest candidates (Table 12). Both tissues are in the top ten in the two proteomic datasets (ranks 3 and 2, respectively) and at rank 15 in the transcriptomic one. This is followed by the pancreas (rank 2 in the HPA transcriptomic and rank 4 in the PtDB proteomic datasets) and the ren (kidney) (rank 9 in the two HPA transcriptomic datasets and rank 4 in the HPA proteomic dataset). However, after normalization by expression entries, the results become less clear (Table 13). Only the colon and pancreas remained among the four highest-ranked tissues overall. Colon retained the highest mean rank across datasets, although it ranked within the top ten only in the HPA proteomic dataset (at rank 5). A similar pattern was observed for the pancreas, which ranked within the top ten only in the HPA transcriptomic dataset (at rank 1).

**Table 12:** Top four tissues based on total aromatic heterocyclic amino acid amount in all three expression datasets

Organ	HPA transcriptomic	HPA proteomic	PtDB proteomic
Colon	15	3	2
Gaster	12	7	5
Pancreas	2	19	4
Ren	9	4	20

**Table 13:** Top four tissues based on total aromatic heterocyclic amino acid amount normalized by number of expression entries in all three expression datasets

Organ	HPA transcriptomic	HPA proteomic	PtDB proteomic
Colon	14	5	16
Intestinum tenue	20	10	5
Pancreas	1	24	12
Duodenum	30	9	6

In contrast, a comparison of the total aromatic heterocyclic loads of all three expression datasets for the organs revealed that the alimentary system is the most consistent candidate among the organs and exhibits the highest rank (rank 1 in all three datasets), followed by the genital and lymphoid systems (both in the top five in all three datasets) (Table 14). This is further supported when the loads are normalized by the number of tissues (ranks 4, 2, and 3, respectively) and expression entries (ranks 4, 3, and 5, respectively), in which case it remains one of the strongest candidates (Tables 15 and 16). Furthermore, it exhibits a medium staining intensity of 0.5 in the MS dataset.

**Table 14:** Top four organs based on total aromatic heterocyclic amino acid amount in all three expression datasets

Organ	HPA transcriptomic	HPA proteomic	PtDB proteomic
Alimentary system	1	1	1
Genital system	3	2	2
Lymphoid system	5	4	3
Nervous system	2	3	7

**Table 15:** Top four organs based on total aromatic heterocyclic amino acid amount normalized by number of tissues in all three expression datasets

Organ	HPA transcriptomic	HPA proteomic	PtDB proteomic
Alimentary system	4	2	3
Respiratory system	9	3	1
Urinary system	5	1	9
Genital system	10	4	2

**Table 16:** Top four organs based on total aromatic heterocyclic amino acid amount normalized by number of expression entries per organ in all three expression datasets

Organ	HPA transcriptomic	HPA proteomic	PtDB proteomic
Alimentary system	4	3	5
Endocrine system	7	4	6
Respiratory system	8	5	4
Sense system	11	1	3

Lastly, as already mentioned, we were unable to find any significant correlations between the enrichment of aromatic heterocycles in organs and their iodine staining intensity. One of the main reasons for this is that organs consist of many proteins and tissues, which leads to a high degree of protein aggregation. This can be explained using the example of muscle: it contains proteins with a high affinity for iodine (e.g., myosin, actin, titin), whereas other proteins do not have this affinity (e.g., collagens). In addition, the grouping in the three expression datasets is insufficient. For example, the two HPA datasets contain only one tissue assigned to muscle, with a low number of proteins (HPA transcriptomics, 15,974; HPA proteomics, 5811), whereas the PtDB dataset shows no expression in this organ. This also applies to other tissues and organs (e.g., ‘cardiovascular system’).

Overall, there is only weak evidence that the iodine staining is due to an enrichment of aromatic heterocyclic amino acids in tissues and organs, which can be attributed to their high aggregation of protein content. However, when considering the raw abundance of aromatic heterocyclic amino acids, the alimentary systems represent a strong candidate for future targeted validation.

## Conclusion and outlook

In this study, we analysed the human proteome at four levels of biological organization to determine whether proteins rich in aromatic heterocyclic amino acids contribute to iodine-based staining of soft tissue. The clearest signal was observed at the protein level, where several proteins exhibited a high concentration or enrichment of aromatic heterocycles and are thus plausible candidates for iodine-sensitive staining.

At the family level, this signal was partially preserved. However, two distinct effects emerged. The average absolute values were mainly determined by families containing very large proteins. Meanwhile, the relative values highlighted families that exhibited genuine enrichment at the sequence level. However, after normalization for sequence length, many structural groups were close to background levels.

At the tissue and organ levels, aromatic heterocyclic enrichment was highly consistent across groups and exhibited only minor variations depending on the expression dataset. After multiple-testing correction, with the exception of three organs in the transcriptomic dataset, neither tissues nor organs showed significant enrichment, and enrichment at the organ level was not robustly associated with the intensity of iodine staining. These results suggest that the signal from proteins rich in aromatic heterocycles is significantly diluted at higher levels of aggregation and is insufficient to explain broad tissue- or organ-level staining patterns.

Analysis of the total aromatic heterocyclic load at the tissue level identified colon and pancreas as the most plausible candidates for iodine staining, both before and after normalization. However, because differentiating tissues for iodine staining was not possible, these findings cannot be validated directly.

At the organ level, the alimentary system emerged as the most consistent candidate across all three expression datasets when total aromatic heterocyclic load was considered. This result remained consistent even after normalization by the number of tissues and expression entries, suggesting that this organ contains a relatively large pool of aromatic heterocycles that can potentially react with iodine.

Overall, our results support the idea that proteins, tissues, and organs rich in aromatic heterocycles could contribute to iodine-based contrast, particularly at the level of individual proteins and specific protein families and organs. However, they also show that higher-level anatomical averages are likely influenced by factors such as tissue composition, protein abundance, and the coarse granularity of available expression and staining data.

Future studies could also examine the effect of iodination on protein stability, in a similar manner to earlier studies of various types of non-enzymatic protein modification, such as bromination and chlorination (Fichtner et al. 2019). Susceptibility scores can be determined for specific amino acids and then aggregated to produce an overall score for a protein. Notably, many of the proteins identified as being of particular interest are localized in the skin and digestive tract, which are frequently exposed to high microbial loads. Given that iodine, particularly in the form of Lugol’s solution (Cooper 2007), is renowned for its antimicrobial properties, this observation provides a compelling basis for further investigation.

## Supplementary Information

Below is the link to the electronic supplementary material.

Supplementary file 1 (zip 31486 KB)

Supplementary file 2 (pdf 903 KB)

## Data Availability

The data supporting this study’s findings are available at https://data.mendeley.com/datasets/nd4ch2vhty.
