# Supplementary material for: Proteomic study for the prediction of μCT imaging with iodine
Source: Histochem Cell Biol. 2026 Sep 25;164(1):89. doi: 10.1007/s00418-026-02532-3 (PMC13615082; doi:10.1007/s00418-026-02532-3)
Supplement: Supplementary file 1 [file 418_2026_2532_MOESM2_ESM.pdf]

Rheumatol Ther
https://doi.org/10.1007/s40744-026-00880-z

ORIGINAL RESEARCH

Real-World Steroid Burden, Treatment Patterns, and Rheumatologists' Perceptions on Advanced Therapy in Giant Cell Arteritis

Anisha B. Dua · Aditi Kadakia · Patrick Zueger · Simran Marwaha · Nathaniel Zerad · Arathi Setty · William Jones · Valerie Devauchelle-Pensec

Received: May 5, 2026 / Accepted: August 7, 2026
© The Author(s) 2026

ABSTRACT

Introduction: Glucocorticoids (GC) are the mainstay of treatment for giant cell arteritis (GCA), but long-term use can cause significant adverse events.
Methods: Rheumatologists from European Union-5 (EU-5) countries (France, Germany, Italy, Spain, and the United Kingdom) and the United States who manage patients with GCA were invited to participate in a cross-sectional retrospective survey (Adelphi Real-World GCA Disease Specific Programmes) between May and October 2024. Rheumatologists completed the surveys based on their experiences managing patients with GCA, including patients' demographic and clinical characteristics, treatment histories, and rates of GC-related adverse events; rheumatologists also shared their opinions on advanced therapies and management strategies. Patients for whom their rheumatologists provided information were invited to self-report their treatment experiences and preferences.
Results: In total, 196 rheumatologists reported on 846 patient cases, of whom 221 (26.1%) patients self-reported their experiences. Among all 846 patients, 63% were female, with a mean age of 71 years and a median of 15.2 months since GCA diagnosis; ≥ 99% received GCs within the last 12 months. For patients diagnosed with GCA < 12, 12–24, and > 24 months ago, median cumulative GC doses over the last 12 months were 4343, 5200, and 2196 mg, respectively. Despite most rheumatologists intending to stop GCs by 24 months, 80% of patients were receiving an average of 8.4 mg/day at 24–< 36 months, with 75% receiving 5.3 mg/day at ≥ 48 months. Most rheumatologists (≥ 90%) agreed that advanced therapies should be given early in GCA treatment, yet only 48% reported prescribing an advanced therapy at any point after diagnosis. Both patients and rheumatologists

UNCORRECTED PROOF

Prior Presentation: These data were previously presented at the *American College of Rheumatology Convergence* in Chicago, IL, USA on October 24–29, 2025.

Supplementary Information The online version contains supplementary material available at https://doi.org/10.1007/s40744-026-00880-z.

A. B. Dua
Northwestern University Feinberg School of Medicine, Chicago, IL, USA

A. Kadakia (✉) · P. Zueger · N. Zerad · A. Setty
AbbVie Inc., Dept GMH1, Blg ABV1-4SE-108-01, 26525 North Riverwoods Blvd., Mettawa, North Chicago, IL 60045, USA
e-mail: aditi.kadakia@abbvie.com

S. Marwaha · W. Jones
Adelphi Real World, Bollington, UK

V. Devauchelle-Pensec
Department of Rheumatology, Centre Hospitalier Universitaire de Brest, Brest, France

reported GC-related adverse events, most commonly weight gain, fatigue, and fluid retention. ***Conclusions***: Despite aiming to taper patients off GCs by 24 months, most patients remained on GCs for ≥ 48 months. Reports of GC-related adverse events highl[illegible]y and effective steroid-s[illegible]n advanced therapies.

Keywords: Advanced therapy; Giant cell arteritis; Glucocorticoids; Interleukin-6; Steroid burden

KEY SUMMARY POINTS

Why carry out this study?

Giant cell arteritis is a medical emergency requiring prompt treatment with high-dose glucocorticoids to prevent permanent damage. However, long-term glucocorticoid use is associated with significant risk of adverse events.

This study sought to determine treatment patterns among patients with giant cell arteritis, specifically glucocorticoid exposure and associated adverse events, as well as use of steroid-sparing advanced therapies, through surveys completed by both rheumatologists and patients.

What was learned from the study?

Despite most rheumatologists intending to taper glucocorticoids by 24 months, 75% of patients were still on glucocorticoids at ≥ 48 months. The most common glucocorticoid-r[illegible]t gain, fatigue, and fluid retention.

Though many rheumatologists support the development and implementation of glucocorticoid-sparing advanced therapies for patients with giant cell arteritis, this study showed that most patients with giant cell arteritis remain on glucocorticoids for years. Further development of glucocorticoid-sparing treatment options, especially those that are more patient-friendly (i.e., oral versus injection), is needed.

INTRODUCTION

Giant cell arteritis (GCA) is the most common form of vasculitis in people aged 50 years and older and is characterized by inflammation of large- and medium-sized blood vessels, including cranial vessels. Patients with GCA typically present with symptoms including headache, scalp tenderness, jaw claudication, and blurred vision; however, clinical manifestations can vary widely, thus GCA can be classified as a heterogeneous disease [1–4]. Morbidity associated with GCA may be substantial, resulting from vascular inflammation and damage or from side effects of long-term glucocorticoid (GC) treatment [2].

The management of GCA typically involves the prompt initiation of high-dose GCs at diagnosis as part of remission induction. Ideally, GCs are tapered within 12 months following

UNCORRECTED PROOF

Journal : Large 40744 | Article No : 880 | Pages : 15 | MS Code : 880 | Dispatch : 12-8-2026

achievement of disease control to limit toxicities [5, 6], including the increased cardiovascular events, osteoporotic fractures, cataracts, and infections that are associated with higher cumulative GC exposure [1, 7–10]. Immunosuppressants may be used as adjunctive therapy in patients with refractory or relapsing disease [11, 12].

Advances in the management of GCA have led to the approval of targeted immunosuppressive agents which have expanded the armamentarium for this disease, providing steroid-sparing treatment approaches that can enable sustained GC-free remission [11, 12]. A study evaluating leflunomide for GCA suggested possible steroid-sparing effects but noted that variability in the available data prohibits meaningful conclusions, so the role of leflunomide in treating GCA may be limited [13]. In 2017, the interleukin-6 (IL-6) receptor antagonist tocilizumab became the first advanced therapy approved for the treatment of GCA after demonstrating higher rates of sustained remission compared to GC monotherapy [12, 14, 15]. A recent study showed that tocilizumab was more effective than methotrexate at preventing relapse after 52 weeks, and clinical guidelines report higher confidence in tocilizumab versus methotrexate to achieve clinically relevant improvements while sparing GCs, suggesting limited value in using methotrexate to treat GCA [16, 17]. In April 2025, the first oral advanced therapy, upadacitinib, a selective and reversible Janus kinase (JAK) inhibitor, was approved by the US Food and Drug Administration and the European Medicines Agency for treatment of adults with GCA after demonstrating superiority in achieving sustained 1-year remission versus GC monotherapy, with maintenance of response demonstrated at 2 years in patients who continued use of upadacitinib [11, 18–21]. These evolving therapeutic approvals have been subsequently incorporated into treatment guidelines to allow for enhanced disease control while sparing GC use [6, 11, 16, 21, 22].

There are a limited number of real-world studies examining GC tapering patterns and GC-related adverse events in patients with GCA [10, 23]. A better understanding of the real-world impact of GC use and physicians' perceptions of advanced therapies may influence the development of new GC-sparing treatments and identify barriers to optimal care. This survey assessed rheumatologists' perceptions of management strategies for patients with GCA, including their actual practice patterns, opinions about advanced therapies, and information on GC-related toxicities experienced by their patients. Patient-reported information regarding GC-related adverse events was also collected via patient surveys, as the patient experience is an important part of evaluating a treatment.

METHODS

Study Design and Participants

Study data were taken from the Adelphi Real World (Cheshire, England) GCA Disease Specific Programmes™ (DSP). This was an independently conducted cross-sectional, retrospective survey of rheumatologists and their patients with GCA in France, Germany, Italy, Spain, and the United Kingdom (European Union-5 [EU-5]), and the United States from May to October 2024 (EU-5: $N = 166$; US: $N = 30$). Rheumatologists were recruited by independent fieldwork agencies who were briefed on the GCA DSP study and the enrollment criteria for both rheumatologists and patients. Eligible rheumatologists had to be actively involved in the management of GCA, having consulted with at least four patients with GCA every month. Rheumatologists could accept or decline study participation and were reimbursed for taking part in the study. Eligible patients were aged ≥ 18 years, had a diagnosis of GCA confirmed by a physician, were actively receiving care from a rheumatologist, and were not currently involved in a clinical trial.

Rheumatologists were surveyed on their approach to patient management and their perceptions of treatment. Patient record forms were completed by the rheumatologists for up to 5 of their next consulting patients with GCA, which recorded patients' diagnostic and treatment journey, symptoms, management and treatment, and concomitant conditions. A list of concomitant conditions considered to be either clinically associated with the diagnosis of GCA

UNCORRECTED PROOF

or important to consider during treatment was derived by Adelphi via a literature search and clinician advice. A full list of the concomitant conditions that were covered in this analysis is included in the Supplementary Material. Rheumatologists were instructed to use their knowledge of the patient and to refer to the patient's medical record to aid in completing the patient record forms. Patients for whom a patient record form was completed were then invited to provide a patient self-completion (PSC) form to capture their own treatment experiences and preferences, including GC use and related adverse events. Patients who completed the PSC questionnaires received no compensation for their time, their participation was entirely voluntary, and they could decline to take part.

Patients provided informed consent for use of their anonymized and aggregated data for research and scientific publication. All data were collected, aggregated, and de-identified prior to receipt. Data collection was performed in line with national market research and privacy regulations according to the European Pharmaceutical Marketing Research Association guidelines, and ethical exemption was sought from the Pearl Institutional Review Board. The study was performed in accordance with the Helsinki Declaration of 1964 and its later amendments.

Outcomes

Perceptions of advanced therapies among rheumatologists in the EU-5 and the United States were recorded. Information gathered assessed rheumatologists' prescribing patterns; their perspective on the preferred attributes of an advanced therapy; and their opinion on when, ideally, advanced therapies should be incorporated into GCA management. Patient demographics and medical histories were reported on patient record forms.

Cumulative and daily oral GC use were recorded using information from the patient record form. Oral GC usage was assessed using a line-based treatment history where a new treatment line was defined by initiation or discontinuation of therapy, change in GC product, initiation or completion of a tapering attempt, or dose increase. All GC doses were converted to their prednisolone equivalent dose separately for each treatment line. Cumulative GC dose was reported as the total GC dose over the observation period, defined as either the 12 months prior to data collection or from the first consultation if this occurred ≤ 12 months prior to data collection. Each treatment line was subsequently summed to generate the total cumulative dose. Details of GC dose calculations are shown in the Supplementary Materials.

Treatment patterns were reported in patients in the EU-5 and the United States. Both rheumatologists and patients were surveyed on GC-related adverse events. Rheumatologists were asked, "*What side effects has the patient experienced in relation to their steroid treatment?*" while patients who completed the PSC questionnaire were asked, "*What side-effect(s) have you experienced?*".

Statistical Analysis

Descriptive statistics were used to summarize patient demographics, treatment patterns, GC dose, GC-related adverse events, and physician perceptions on the use of advanced therapies. Continuous variables were reported as means, medians, ranges, and standard deviations and categorical variables were reported as frequency distributions. All analyses were conducted using Stata Statistical Software: Release 18 (College Station, TX, USA).

RESULTS

Demographics and Patient History

From the 196 rheumatologists surveyed, data on a total of 846 patients with GCA were included in the analysis (EU-5: $N = 646$; US: $N = 200$) of whom, 221 (26.1%) patients completed a PSC and 625 did not complete a PSC (73.9%; Table 1). Of these patients, 63% were female, 94% were white, and the mean (± standard deviation) age was 71 ± 9.6 years. The median time since first diagnosis of GCA was 14.2 (interquartile range: 26.7) months. Most patients (77%)

UNCORRECTED PROOF

Journal : Large 40744	Article No : 880	Pages : 15	MS Code : 880	Dispatch : 12-8-2026

Table 1 Demographics and patient history at time of data collection

Characteristic	Total patient sample<br>$N = 846$
Female, *n* (%)	530 (63)
Race (White), *n* (%)	795 (94)
Age [years], mean (SD)	71.3 (9.6)
Country, *n* (%)	
France	133 (16)
Germany	120 (14)
Italy	144 (17)
Spain	120 (14)
United Kingdom	129 (15)
United States	200 (24)
Time from diagnosis [months], median (IQR)	14.2 (26.7)
Patients diagnosed by a rheumatologist, *n* (%)	654 (77)
Test used to diagnose GCA, *n* (%)	
Unilateral temporal artery biopsy	460 (54)
Temporal artery ultrasound	426 (50)
Computed tomography	103 (12)
Positron emission tomography	91 (11)
Most common concomitant autoimmune comorbidities, *n* (%)	
Polymyalgia rheumatica	348 (41)
Psoriasis	27 (3)
Inflammatory bowel disease	23 (3)
Rheumatoid arthritis	22 (3)
Atopic dermatitis	20 (2)
Most common concomitant non-autoimmune comorbidities, *n* (%)	
Osteoporosis	172 (20)
Diabetes with chronic complications	133 (16)
Anxiety	115 (14)
Depression	102 (12)
Obesity	80 (9)

UNCORRECTED PROOF

Journal : Large 40744 | Article No : 880 | Pages : 15 | MS Code : 880 | Dispatch : 12-8-2026

Table 1 continued

**Characteristic**	**Total patient sample** *N* = 846
Cumulative glucocorticoid dose [mg] over 12-month baseline by time since diagnosis, median (IQR) [min–max]	
< 12 months	4343 (5327) [0–46,150]
12–24 months	5200 (6954) [0–24,280]
> 24 months	2196 (4342) [0–36,600]
Cumulative glucocorticoid dose [mg/day] at survey date by time since diagnosis, median (IQR)	
< 12 months	16.3 (35.0)
12–24 months	5.0 (8.0)
> 24 months	5.0 (3.5)
Non-steroidal treatments received at data collection, *n* (%)	
Leflunomide	18 (2)
Methotrexate	198 (23)
Other non-biologic immunosuppressive therapy	8 (1)
Abatacept	7 (1)
Sarilumab	15 (2)
Tocilizumab	220 (26)
Ustekinumab	5 (1)
Other biologic	3 (< 1)
Baricitinib	4 (< 1)
Upadacitinib	7 (1)
Other JAK inhibitor	0 (0)
Other treatments	2 (< 1)
Not receiving a non-GC treatment	404 (48)

GC glucocorticoid, *GCA* giant cell arteritis, *IQR* interquartile range, *JAK* Janus kinase, *SD* standard deviation

UNCORRECTED PROOF

were diagnosed by a rheumatologist either by unilateral temporal artery biopsy (*n* = 460) and/or temporal artery ultrasound (*n* = 426). Polymyalgia rheumatica occurred in 41% (*n* = 348) of patients surveyed. The most common non-autoimmune comorbidity was osteoporosis (20%), followed closely by diabetes with chronic complications (16%). At the time of data collection,

Journal : **Large 40744**	Article No : **880**	Pages : **15**	MS Code : **880**	Dispatch : **12-8-2026**

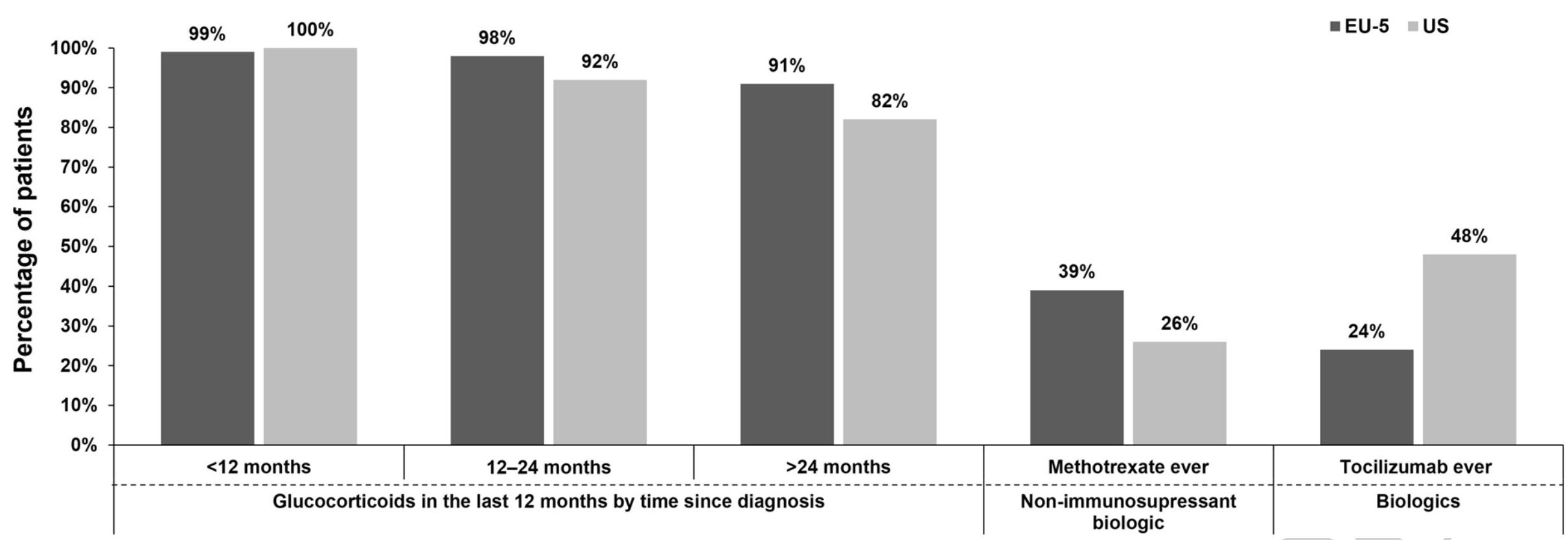

Fig. 1 Treatments received by patients in the EU-5 and the United States. *EU-5* European Union-5

52% ($n=442$) of patients were receiving a non-GC treatment.

Treatment Patterns in the EU-5 and United States

Most patients surveyed had received GCs within the last 12 months, irrespective of time since diagnosis (Fig. 1). Among patients diagnosed < 12 months, 12–24 months, and > 24 months ago, respectively, 99%, 98%, and 91% of patients in the EU-5 and 100%, 92%, and 82% in the United States had used GCs in the last 12 months.

Aside from GCs, tocilizumab and methotrexate were the most commonly used treatments. Tocilizumab was used by 48% of patients in the United States and 24% of patients in the EU-5, whereas methotrexate was used by 39% of patients in the EU-5 and 26% of patients in the United States. Among those who had ever received targeted therapies, the median time from diagnosis to receiving tocilizumab was 3.8 months in United States and 6.0 months in EU-5 countries; the median time from diagnosis to receiving methotrexate was approximately 5 months regardless of location.

Oral GC Use by Disease Duration

The median (interquartile range [IQR]) reported cumulative GC doses over the previous 12 months were 4343 (5327), 5200 (6954), and 2196 (4342) mg for patients diagnosed < 12, 12–24, and > 24 months ago, respectively (Table 1). The median (IQR) current GC dose was 16.3 (35.0), 5.0 (8.0), and 5.0 (3.5) mg/day for patients diagnosed < 12, 12–24, and > 24 months ago, respectively. Most rheumatologists (87%) stated that they aim to taper patients to low-dose GCs by 12 months post-diagnosis and 81% stated that they aim to taper patients off GCs completely by 24 months post-diagnosis. In this analysis, 90% of patients were receiving GCs at 9 to < 12 months post-diagnosis (mean dose: 13.1 mg/day; Fig. 2); 80% and 75% of patients were still receiving GCs at 24 to < 36 months and ≥ 48 months postdiagnosis, respectively, with mean doses of 8.4 and 5.3 mg/day, respectively.

GC-Related Adverse Events

= Rheumatologists reported that the most frequent GC-related adverse events were weight gain (35%), fatigue/tiredness (25%), and fluid retention (19%; Fig. 3A). Patients who completed a PSC reported that the most frequent adverse events were weight gain (38%), fatigue (26%), and fluid retention (23%; Fig. 3B).

Rheumatologists' and Patients' Perspectives on Advanced Therapies

Of the 196 rheumatologists surveyed (EU-5: $N=166$; US: $N=30$), when asked how important they perceived each attribute to be for an advanced therapy, more than 90% noted that

UNCORRECTED PROOF

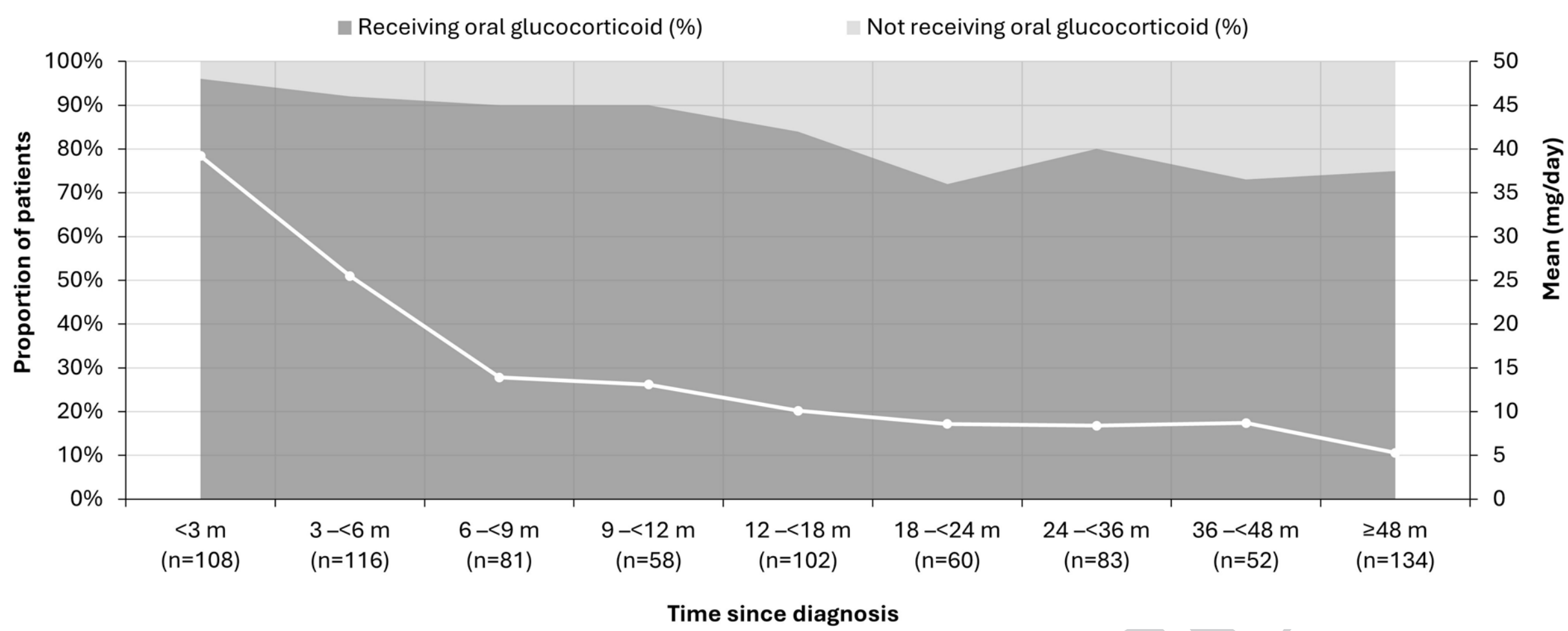

Fig. 2 Oral glucocorticoid dose received by disease duration. *m* months

the following attributes were very or extremely important: support maintenance of remission (EU-5: 99%; US: 96%); increase achievement of GC-free remission (EU-5: 95%; US: 97%); reduce high-dose/cumulative GC use (EU-5: 92%; US: 96%); allow for GC cessation (EU-5: 93%; US: 97%); be safe and tolerable (EU-5: 97%; US: 100%); and have features that support patient adherence (e.g., convenient dosing or administration; EU-5: 90%; US: 93%; Table 2). The majority of rheumatologists also reported convenient administration as an important attribute of advanced therapies (EU-5: 65%; US: 84%). Similarly, 75% of patients stated that they preferred receiving treatments orally rather than by injection or infusion.

Almost all rheumatologists (EU-5, 90%; US, 93%) reported that advanced therapies should be given early in GCA treatment to improve outcomes and reduce GC toxicity. Most rheumatologists, particularly in the United States, reported that limited choices in the administration routes of advanced therapies are a barrier to widespread use (EU-5, 54%; US, 80%). They agreed / strongly agreed that the launch of new treatments would result in earlier use of advanced therapies (EU-5, 84%; US, 100%) and that they would use advanced therapies more often if there were more options available (EU-5, 75%; US, 97%).

DISCUSSION

In this retrospective survey, most patients with GCA in the EU-5 and United States had used GCs in the last 12 months, irrespective of time since diagnosis. The majority of rheumatologists aimed to taper GCs to low doses within 1 year and taper off completely by 2 years postdiagnosis. Despite this, the present survey reveals that most patients (80%) were on an average daily GC dose of 8.4 mg after 2 years, and two-thirds of patients continued on low-dose GCs (5.3 mg/day) at 4 years or more after their diagnosis. Overall, this retrospective survey suggests a possible gap in the funding or implementation of advanced therapeutics given the discrepancy in intentions, practice patterns, and clinical guidelines.

Other real-world studies have also reported similarly long durations of GC treatment in patients with GCA. In a German retrospective database review of patients with GCA and/or polymyalgia rheumatica, one-quarter of all patients studied had used GCs for ≥ 3 years [24]. Three US retrospective studies reported that more than two-thirds of patients with GCA and/or polymyalgia rheumatica were still receiving prednisone ≥ 2 years after starting treatment and more than one-third of patients had evidence

UNCORRECTED PRO

Journal : Large 40744 | Article No : 880 | Pages : 15 | MS Code : 880 | Dispatch : 12-8-2026

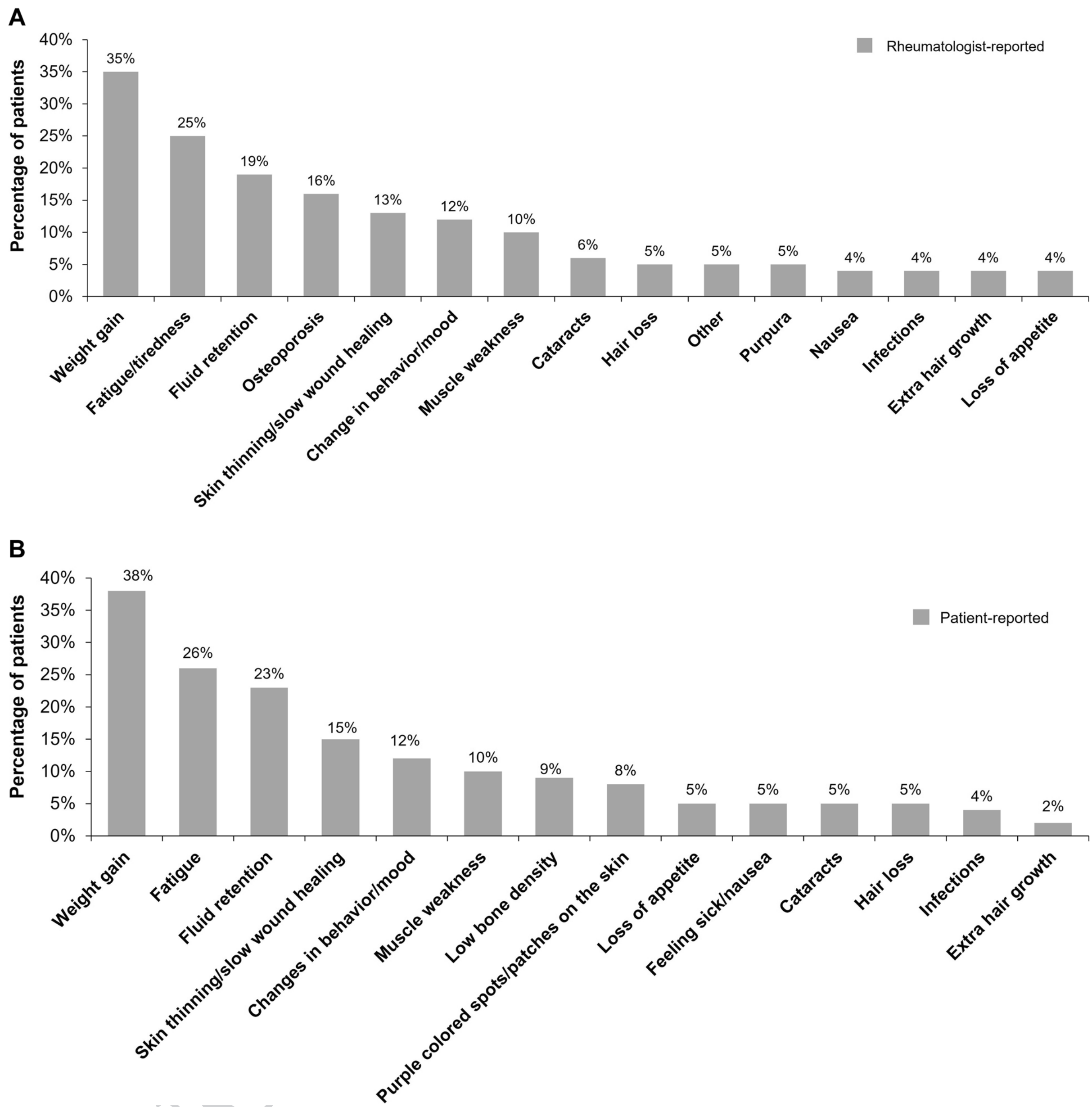

Fig. 3 Glucocorticoid-related AEs reported by rheumatologists and/or patients among **A** all patients and **B** patients who self-reported AEs. **A** Rheumatologist-reported glucocorticoid-related AEs ($N = 846$). **B** Patient-reported glucocorticoid-related AEs ($N = 221$). *AE* adverse event

of GC use for up to 3 years, which persisted at 5 years following diagnosis [10, 25, 26].

There is a lack of clarity around the maximum length of time over which GCs should be administered in GCA, with current guidelines not defining a specific duration of treatment [6, 16, 22]. EULAR guidelines recommended high-dose GC therapy for initial induction of remission in active GCA, tapering once the disease is controlled, and suggest that doses should be reduced to ≤ 5 mg/day after 1 year of treatment [6]. They additionally note that the typical duration of GC therapy may be more than 2 years [5]. The British Society of Rheumatology guidelines state

Journal : Large 40744	Article No : 880	Pages : 15	MS Code : 880	Dispatch : 12-8-2026

Table 2 Rheumatologists' perceptions on advanced therapies

	EU-5 *N* = 166	United States *N* = 30
How important do you perceive each of the following attributes to be for an advanced treatment indicated for GCA?[a]		
Maintains remission		
Somewhat important	1 (1%)	1 (3%)
Very important	60 (36%)	10 (33%)
Extremely important	105 (63%)	19 (63%)
Maintains glucocorticoid-free remission		
Somewhat important	9 (5%)	1 (3%)
Very important	53 (32%)	12 (40%)
Extremely important	104 (63%)	17 (57%)
Reduces cumulative glucocorticoid dose		
Somewhat important	13 (8%)	1 (3%)
Very important	72 (43%)	10 (33%)
Extremely important	81 (49%)	19 (63%)
Allows for stopping glucocorticoids altogether		
Somewhat important	11 (7%)	1 (3%)
Very important	64 (39%)	9 (30%)
Extremely important	89 (54%)	20 (67%)
Safety/tolerability		
Somewhat important	5 (3%)	0 (0%)
Very important	68 (41%)	9 (30%)
Extremely important	93 (56%)	21 (70%)
Patient compliance		
Somewhat important	16 (10%)	2 (7%)
Very important	92 (55%)	13 (43%)
Extremely important	58 (35%)	15 (50%)
Physician's opinions on each statement regarding the use of advanced therapies[b]		
Advanced therapies should be used early in patients with GCA to significantly improve outcomes and reduce glucocorticoid toxicity		
Disagree/strongly disagree	16 (10%)	2 (7%)
Agree/strongly agree	150 (90%)	28 (93%)

UNCORRECTED PROOF

Table 2 continued

	EU-5 $N = 166$	United States $N = 30$
Lack of variety in advanced treatment routes of administration is a barrier to more widespread use of advanced therapy		
Disagree/strongly disagree	77 (46%)	6 (20%)
Agree/strongly agree	89 (54%)	24 (80%)
The launch of new treatments results in earlier use of advanced therapy		
Disagree/strongly disagree	26 (16%)	0 (0%)
Agree/strongly agree	140 (84%)	30 (100%)
I would use advanced therapies more often if there were more options		
Disagree/strongly disagree	42 (25%)	1 (3%)
Agree/strongly agree	124 (75%)	29 (97%)

[a]No more than one rheumatologist answered "of no importance" for any given question

[b]Including biologics, JAK inhibitors

EU-5 European Union-5, *GCA* giant cell arteritis, *JAK* Janus kinase

that GC dose should be tapered to zero over 12–18 months, providing there is no return of symptoms [22]. The American College of Rheumatology/Vasculitis Foundation guideline also recommends tapering of GCs to avoid prolonged use and reduce toxicity, but acknowledges that longer-term use may be required to avoid disease flares in some patients [16, 22].

The present study confirms that, despite guideline recommendations to minimize length of GC treatment, many physicians continue to treat patients with low GC doses over extended periods. It has been shown that chronic low-dose GC therapy can result in adverse events [9, 27]. In our study, although rheumatologists stated they intended to taper to low GC doses within 1 year of diagnosis, and completely off within 2 years, 75% of patients were still receiving GCs ≥ 48 months after diagnosis. Adverse events related to GC use were self-reported by more than half of patients, while, according to rheumatologists, almost two-thirds of patients experienced at least one GC-related adverse event. Despite the increased risk of GC-related side effects, prolonged GC use may reflect the fact that up to 80% of patients will experience at least one relapse with GC monotherapy over the course of their disease, leading to additional GC therapy, and highlights the need for therapies with greater effectiveness as well as GC-sparing ability [28, 29]. Moreover, approximately 40% of patients are unable to discontinue GCs without relapsing [29, 30]. These data highlight the careful balance needed to maintain disease control while minimizing the risk of GC adverse events in the long-term treatment of GCA.

Toxicities and side effects of GC use, both in the short and long term, are well described in the literature. One survey study in France ($N = 90$) found that almost all patients had at least one GC-related adverse event, commonly psychological disorders, cutaneous changes, and muscle loss, with vision loss and bone fractures being common among patients with > 18 months of GC-use [8]. In a retrospective review of 139 consecutive patients with GCA, 94% of patients presented at least one GC-related adverse event, most commonly infections and metabolic events, which were associated with cumulative GC doses > 9 mg/day and longer GC duration [7]. Similarly, a real-world retrospective database analysis of patients with GCA in the United States ($N = 4804$) and the United Kingdom ($N = 3973$) demonstrated that an increased cumulative GC exposure was associated with an increased risk of adverse events,

UNCORRECTED PROOF

Journal : Large 40744 | Article No : 880 | Pages : 15 | MS Code : 880 | Dispatch : 12-8-2026

most commonly hypertension, eye-related disorders (e.g., glaucoma, cataracts), bone-related events (e.g., osteoporosis, fracture), and glucose tolerance conditions [9]. Together, these data underline the importance of carefully monitoring GC-related adverse events during treatment and highlight the need to reduce GC use by encouraging the use of GC-sparing strategies.

In our study, most rheumatologists believed that GC-sparing therapies, such as biologics and JAK inhibitors, should be given early in the treatment of GCA to improve outcomes and avoid GC toxicity. This notion is supported by the American College of Rheumatology, which recommends the use of an advanced therapy at the time of diagnosis to allow for more rapid GC reduction and efficacy [16]. Likewise, EULAR recommends first-line use of GC-sparing therapies in patients with a high risk of GC-related AEs or with relapsing/refractory disease [6]. Despite this, the current study showed that only 24–48% of patients in the EU-5 and US had ever received an advanced therapy. Further data are needed to understand the barriers preventing greater use of advanced therapies in this population, as well as to identify specific patient populations that may receive the greatest benefit from early treatment with advanced therapies.

Attributes of advanced therapies considered important by rheumatologists included efficacy (i.e., maintenance of remission/achievement of GC-free remission), reduction in long-term GC use, and safety/tolerability. The recent approval of upadacitinib for the treatment of adults with GCA has expanded GC-sparing treatment [18]. Unlike tocilizumab, which is administered subcutaneously or intravenously, upadacitinib is an oral therapy and may offer greater convenience for patients. Rheumatologists identified convenient administration as a key attribute for advanced therapies and 75% of patients in this study preferred oral treatments over injections or infusions. Advanced therapies that align with both provider and patient preferences may improve adoption of these therapies, reduce GC-related adverse events, and increase patient satisfaction. Further research is needed to better understand treatment decisions as new treatment options become available.

Limitations of the present study include the retrospective design. Methodological limitations of such surveys include the potential for response or sampling bias, context-blindness, or misunderstanding of questions. Moreover, there may be limited depth of detail possible in responses, meaning that incomplete information is captured, for example relating to other treatments or aspects of care. Interpretation of the results should also consider the industry-sponsored nature of this analysis. Furthermore, because the study was conducted only in EU-5 countries and the United States, the results may not reflect other geographic areas. However, our findings align with previous real-world analyses, underscoring the need for effective, GC-sparing therapies in the treatment of GCA, which can reduce the need for long-term GC use and associated adverse events [26]. A key strength of this analysis is that not only did the survey capture rheumatologists' intentions for GCA management, but it also recorded their actual clinical practices and surveyed a large number of patients regarding their perspectives on and experience with GCA management.

CONCLUSIONS

Although most rheumatologists aim to taper patients off GCs completely by 24 months, 75% of patients were still on GC treatment 4 years after diagnosis. Both rheumatologists and patients reported several adverse events associated with GC use, and rheumatologists reported the need for more effective GC-sparing advanced treatment options. In this survey, most rheumatologists agreed that advanced therapies should be given early; however, less than a quarter of patients in EU-5 countries have ever received an advanced therapy. These data suggest that further research into identifying patient phenotypes and improving treatment timing to enhance the benefits of advanced therapies is needed. In conclusion, optimizing treatment strategies beyond GCs is critical, and greater real-world adoption of advanced therapies could close the gap between guideline recommendations and clinical practice.

UNCORRECTED PROOF

Journal : Large 40744 | Article No : 880 | Pages : 15 | MS Code : 880 | Dispatch : 12-8-2026

Author Contributions. Substantial contributions to conception and design: Aditi Kadakia, Simran Marwaha, William Jones. Acquisition of data: Simran Marwaha, William Jones. Data analysis: William Jones. Interpretation of data: Anisha B. Dua, Aditi Kadakia, Patrick Zueger, Simran Marwaha, Nathaniel Zerad, Arathi Setty, William Jones, Valerie Devauchell-Pensec. Involved in drafting/revising critically for intellectual content: Anisha B. Dua, Aditi Kadakia, Patrick Zueger, Simran Marwaha, Nathaniel Zerad, Arathi Setty, William Jones, Valerie Devauchell-Pensec.

Funding. Data collection was undertaken by Adelphi Real World as part of an independent survey, entitled the Adelphi Giant Cell Arteritis Disease Specific Programme (DSP). The DSP is a wholly owned Adelphi product and is the intellectual property of Adelphi Real World. The analysis described here used survey data from the Adelphi GCA DSP. AbbVie was one of multiple subscribers to the DSP and did not influence the original survey through either contribution to the design of questionnaires or data collection. Adelphi analyzed the data for AbbVie. AbbVie participated in the research, analysis design, interpretation of the data, review, and approval. All authors had access to the relevant data and participated in the drafting, review, and approval of the manuscript. No honoraria or payments were made for authorship. The journal's Rapid Service Fee was funded by AbbVie.

Data Availability. The datasets generated during and/or analyzed during the current study are available from the corresponding author on reasonable request.

Declarations

Conflict of Interest. A.B. Dua has received consulting fees from AbbVie, Amgen, AZ, GSK, Novartis, and Sanofi; has participated in clinical trials or received grant support from Amgen, Novartis, and Rheumatology Research Foundation; and was on the Board of Directors for Vasculitis Foundation and Rheumatology Research Foundation. A. Kadakia, P. Zueger, N. Zerad, and A. Setty are employees of AbbVie and may own stock or stock options. S. Marwaha is a former employee of Adelphi Real World and current employee of Research Partnership. W. Jones is an employee of Adelphi Real World. V. Devauchelle-Pensec has received grants from Fresenius and consultancy fees from AbbVie and Novartis.

Ethical Approval. Patients provided informed consent for use of their anonymized and aggregated data for research and scientific publication. All data were collected, aggregated, and de-identified prior to receipt. Data collection was performed in line with national market research and privacy regulations according to the European Pharmaceutical Marketing Research Association guidelines, and ethical exemption was sought from the Pearl Institutional Review Board. The study was performed in accordance with the Helsinki Declaration of 1964 and its later amendments.

Medical Writing, Editorial, and Other Assistance. Medical writing services were provided by Samantha D. Francis Stuart, PhD, of Avalere Health, Ltd., and funded by AbbVie.

Open Access. This article is licensed under a Creative Commons Attribution-NonCommercial 4.0 International License, which permits any non-commercial use, sharing, adaptation, distribution and reproduction in any medium or format, as long as you give appropriate credit to the original author(s) and the source, provide a link to the Creative Commons licence, and indicate if changes were made. The images or other third party material in this article are included in the article's Creative Commons licence, unless indicated otherwise in a credit line to the material. If material is not included in the article's Creative Commons licence and your intended use is not permitted by statutory regulation or exceeds the permitted use, you will need to obtain permission directly from the copyright holder. To view a copy of this licence, visit http://creativecommons.org/licenses/by-nc/4.0/.

UNCORRECTED PROOF

REFERENCES

1. Curtis JR, Conrad DM, Krueger WS, Gara AP, Winthrop KL. Real-world data on the use of the Shingrix vaccine among patients with inflammatory arthritis and risk of cardiovascular events following herpes zoster. Arthritis Res Ther. 2025;27(1):108.

2. Li KJ, Semenov D, Turk M, Pope J. A meta-analysis of the epidemiology of giant cell arteritis across time and space. Arthritis Res Ther. 2021;23(1):82.

3. Ponte C, Grayson PC, Robson JC, et al. 2022 American College of Rheumatology/EULAR Classification Criteria for giant cell arteritis. Ann Rheum Dis. 2022;81(12):1647–53.

4. Shiomi M, Watanabe R, Ishihara R, et al. Uncovering the new biology of giant cell arteritis to guide therapeutic strategies. J Clin Med. 2025. https://doi.org/10.3390/jcm14186350.

5. de Boysson H, Devauchelle-Pensec V, Agard C, et al. French protocol for the diagnosis and management of giant cell arteritis. Rev Med Interne. 2025;46(1):12–31.

6. Mukhtyar CB, Nikiphorou E, Bartoletti A, et al. 2025 EULAR recommendations for the management of polymyalgia rheumatica and primary large vessel vasculitis. Ann Rheum Dis. 2025. https://doi.org/10.1016/j.ard.2026.06.009.

7. Castan P, Dumont A, Deshayes S, et al. Impact of glucocorticoid cumulative doses in a real-life cohort of patients affected by giant cell arteritis. J Clin Med. 2022. https://doi.org/10.3390/jcm11041034.

8. de Boysson H, Barakat C, Dumont A, et al. Tolerance of glucocorticoids in giant cell arteritis: a study of patient-reported adverse events. Rheumatology (Oxford). 2022;61(9):3567–75.

9. Gale S, Wilson JC, Chia J, et al. Risk associated with cumulative oral glucocorticoid use in patients with giant cell arteritis in real-world databases from the USA and UK. Rheumatol Ther. 2018;5(2):327–40.

10. Curtis JR, Kruzikas DT, Romero A, et al. Real-world analysis of patient characteristics, adverse events, and economic burden among patients with giant cell arteritis treated with glucocorticoids. Rheumatol Ther. 2025;12(5):835–54.

11. Blockmans D, Penn SK, Setty AR, et al. A phase 3 trial of upadacitinib for giant-cell arteritis. N Engl J Med. 2025;392(20):2013–24.

12. Stone JH, Tuckwell K, Dimonaco S, et al. Trial of tocilizumab in giant-cell arteritis. N Engl J Med. 2017;377(4):317–28.

13. Zhu LM, Mendel A, Ross C, Makhzoum JP. The effectiveness and safety of leflunomide in the treatment of giant cell arteritis: a systematic review and meta-analysis. Rheumatol Adv Pract. 2025;9(4):rkaf128.

14. Genentech Inc. Actemra (tocilizumab) prescribing information. 2025.

15. Roche Pharma AG. Roactemra (tocilizumab) summary of product characteristics. 2025.

16. Maz M, Chung SA, Abril A, et al. 2021 American College of Rheumatology/Vasculitis Foundation guideline for the management of giant cell arteritis and Takayasu arteritis. Arthritis Rheumatol. 2021;73(8):1349–65.

17. Samson M, Bourredjem A, Outh R, et al. Methotrexate versus tocilizumab for treatment of giant cell arteritis (METOGiA trial): a multicenter, randomized, controlled trial [abstract]. Arthritis Rheumatol. 2025;77(suppl 9.). AQ2

18. AbbVie. RINVOQ® (upadacitinib) receives U.S. FDA approval for giant cell arteritis (GCA)

19. AbbVie Deutschland GmbH & Co. RINVOQ summary of product characteristics. 2025. AQ3

20. AbbVie I. RINVOQ (upadacitinib) prescribing information. 2025.

21. Schmidt W, Setty AR, Dejaco C, et al. Efficacy and safety of upadacitinib in giant cell arteritis: 2-year results from the re-randomized, double-blind select-GCA phase 3 trial [abstract]. Arthritis Rheumatol. 2025;77.

22. Mackie SL, Dejaco C, Appenzeller S, et al. British society for rheumatology guideline on diagnosis and treatment of giant cell arteritis. Rheumatology (Oxford). 2020;59(3):e1–23.

23. Mahr A, Hachulla E, de Boysson H, et al. Presentation and real-world management of giant cell arteritis (ARTEMIS study). Front Med (Lausanne). 2021;8:732934.

24. Albrecht K, Huscher D, Buttgereit F, et al. Long-term glucocorticoid treatment in patients with polymyalgia rheumatica, giant cell arteritis, or both diseases: results from a national rheumatology database. Rheumatol Int. 2018;38(4):569–77.

25. Chandran A, Udayakumar PD, Kermani TA, et al. Glucocorticoid usage in giant cell arteritis over

UNCORRECTED PROOF

Journal : Large 40744	Article No : 880	Pages : 15	MS Code : 880	Dispatch : 12-8-2026

six decades (1950 to 2009). Clin Exp Rheumatol. 2015;33(2 Suppl 89):S-98-102.

26. Craig G, Knapp K, Salim B, Mohan SV, Michalska M. Treatment patterns, disease burden, and outcomes in patients with giant cell arteritis and polymyalgia rheumatica: a real-world, electronic health record-based study of patients in clinical practice. Rheumatol Ther. 2021;8(1):529–39.

27. Strehl C, Bijlsma JW, de Wit M, et al. Defining conditions where long-term glucocorticoid treatment has an acceptably low level of harm to facilitate implementation of existing recommendations: viewpoints from an EULAR Task Force. Ann Rheum Dis. 2016;75(6):952–7.

28. Alba MA, Garcia-Martinez A, Prieto-Gonzalez S, et al. Relapses in patients with giant cell arteritis: prevalence, characteristics, and associated clinical findings in a longitudinally followed cohort of 106 patients. Medicine (Baltimore). 2014;93(5):194–201.

29. Tsalapaki C, Lazarini A, Argyriou E, et al. Glucocorticoid discontinuation rate and risk factors for relapses in a contemporary cohort of patients with giant cell arteritis. Rheumatol Int. 2024;44(4):603–10.

30. Labarca C, Koster MJ, Crowson CS, et al. Predictors of relapse and treatment outcomes in biopsy-proven giant cell arteritis: a retrospective cohort study. Rheumatology (Oxford). 2016;55(2):347–56.

UNCORRECTED PROOF

Journal:	**40744**
Article:	**880**

Author Query Form

Please ensure you fill out your response to the queries raised below and return this form along with your corrections

Dear Author

During the process of typesetting your article, the following queries have arisen. Please check your typeset proof carefully against the queries listed below and mark the necessary changes either directly on the proof/online grid or in the 'Author's response' area provided below

Query	Details Required	Author's Response
AQ1	Kindly check and confirm whether the affiliation 2 is processed correctly.	
AQ2	AUTHOR: As References 18. and 31. are same, we have deleted the duplicate reference and renumbered accordingly. Please check and confirm.	
AQ3	Kindly provide complete details for Refs. [18-20].	
